# A fusion analytic framework for investigating functional brain connectivity differences using resting-state fMRI

**DOI:** 10.3389/fnins.2024.1402657

**Published:** 2024-12-11

**Authors:** Yeseul Jeon, Jeong-Jae Kim, SuMin Yu, Junggu Choi, Sanghoon Han

**Affiliations:** ^1^Department of Statistics, Texas A&M University, College Station, TX, United States; ^2^Graduate Program in Cognitive Science, Yonsei University, Seoul, Republic of Korea; ^3^Department of Psychology and Neuroscience, Duke University, Durham, NC, United States; ^4^Cancer Biology, Cleveland Clinic, Lerner Research Institute, Cleveland, OH, United States; ^5^Department of Psychology, Yonsei University, Seoul, Republic of Korea

**Keywords:** fMRI, ADNI, functional connectivity network, deep learning, Latent Space Item-Response Model

## Abstract

**Introduction:**

Functional magnetic resonance imaging (fMRI) data is highly complex and high-dimensional, capturing signals from regions of interest (ROIs) with intricate correlations. Analyzing such data is particularly challenging, especially in resting-state fMRI, where patterns are less identifiable without task-specific contexts. Nonetheless, interconnections among ROIs provide essential insights into brain activity and exhibit unique characteristics across groups.

**Methods:**

To address these challenges, we propose an interpretable fusion analytic framework to identify and understand ROI connectivity differences between two groups, revealing their distinctive features. The framework involves three steps: first, constructing ROI-based Functional Connectivity Networks (FCNs) to manage resting-state fMRI data; second, employing a Self-Attention Deep Learning Model (Self-Attn) for binary classification to generate attention distributions encoding group-level differences; and third, utilizing a Latent Space Item-Response Model (LSIRM) to extract group-representative ROI features, visualized on group summary FCNs.

**Results:**

We applied our framework to analyze four types of cognitive impairments, demonstrating their effectiveness in identifying significant ROIs that contribute to the differences between the two disease groups. The results reveal distinct connectivity patterns and unique ROI features, which differentiate cognitive impairments. Specifically, our framework highlighted group-specific differences in functional connectivity, validating its capability to capture meaningful insights from high-dimensional fMRI data.

**Discussion:**

Our novel interpretable fusion analytic framework addresses the challenges of analyzing high-dimensional, resting-state fMRI data. By integrating FCNs, a Self-Attention Deep Learning Model, and LSIRM, the framework provides an innovative approach to discovering ROI connectivity disparities between groups. The attention distribution and group-representative ROI features offer interpretable insights into brain activity patterns and their variations among cognitive impairment groups. This methodology has significant potential to enhance our understanding of cognitive impairments, paving the way for more targeted therapeutic interventions.

## 1 Introduction

Functional magnetic resonance imaging (fMRI) data, particularly resting-state fMRI (rs-fMRI), is inherently complex and high-dimensional. This complexity results in correlated matrices that capture signals from regions of interest (ROIs) in the brain at each time point. Several attempts have been made to analyze fMRI data to understand the roles of ROIs in specific tasks or symptoms (Santana et al., 2022; Wang et al., 2016). Comparing ROIs in fMRI data from different tasks has been one approach to understanding their mechanisms and identifying group differences (Li et al., 2018; Lee and Lee, 2020). However, interpreting which features of ROI connections differentiate between groups has proven challenging for previous studies (Smith et al., 2010; Gates and Molenaar, 2012). There are two main reasons for this difficulty: First, the high-dimensional and correlated nature of fMRI datasets makes it difficult to apply standard statistical models, which typically assume that the data is independent and identically distributed. The complex interactions and dependencies among ROIs in fMRI data make this independence assumption unrealistic, leading to biased or inaccurate interpretations (Smith et al., 2010, 2011). Second, identifying distinctive ROI connectivity that represents group differences is challenging because of the noise introduced by individual effects. Each fMRI dataset corresponds to an independent subject, and the inherent variability and noise in individual effects can obscure the true underlying patterns that distinguish different groups or conditions (Dubois and Adolphs, 2016).

To address these limitations, we propose a novel analytic framework that integrates a deep learning-based classification model with a statistical model, while providing visual interpretation through the functional connectivity networks (FCNs) of ROIs to offer intuitive insights. Since deep learning models are well-known for handling high-dimensional correlated structured data (Du et al., 2022), they are appropriate to apply fMRI data that exhibit complex interactions and dependencies among ROIs. In this study, we utilize a Self-Attention Deep Learning Model (Self-Attn). Self-Attn employs the self-attention mechanism (Vaswani et al., 2017), which is capable of handling correlated structured data and effectively learning adjacency connections (Chen et al., 2018; Zheng et al., 2019; Sun et al., 2020). This enables us to capture intricate connectivity patterns between ROIs in fMRI data. The self-attention mechanism provides an attention distribution for the ROIs, indicating how Self-Attn learns the relationships in the structured input data. Each row in the attention distribution reflects the likelihood of how a specific ROI relates to other ROIs. If the classification accuracy is sufficient, the output of the attention distribution for each subject's ROIs is a reliable source for identifying the ROI connections that distinguish different groups.

However, it is still challenging to identify which ROI connections differentiate groups by manually comparing these distributions. To address this, we analyze the ROIs' attention distribution using the Latent Space Item-Response Model (LSIRM)(Jeon Y. et al., 2021), a statistical network model. We interpret the attention distribution as an item-response matrix (Embretson and Reise, 2013), where ROIs represent items and subjects represent respondents. To the best of our knowledge, there has been no prior research that analyzes the ROIs' attention distribution in the context of statistical network models. Here, the LSIRM estimates relationships between respondents and ROIs by modeling the probabilities of positive responses (connections). This estimation allows our framework to select group-representative ROIs that are consistently shown as meaningful ROIs across all individuals within each group. As a result, our framework effectively tackles the previous challenge of noise caused by individual variations. These distinctive ROI connections are then visualized on the group summary FCN.

**Figure 1:**
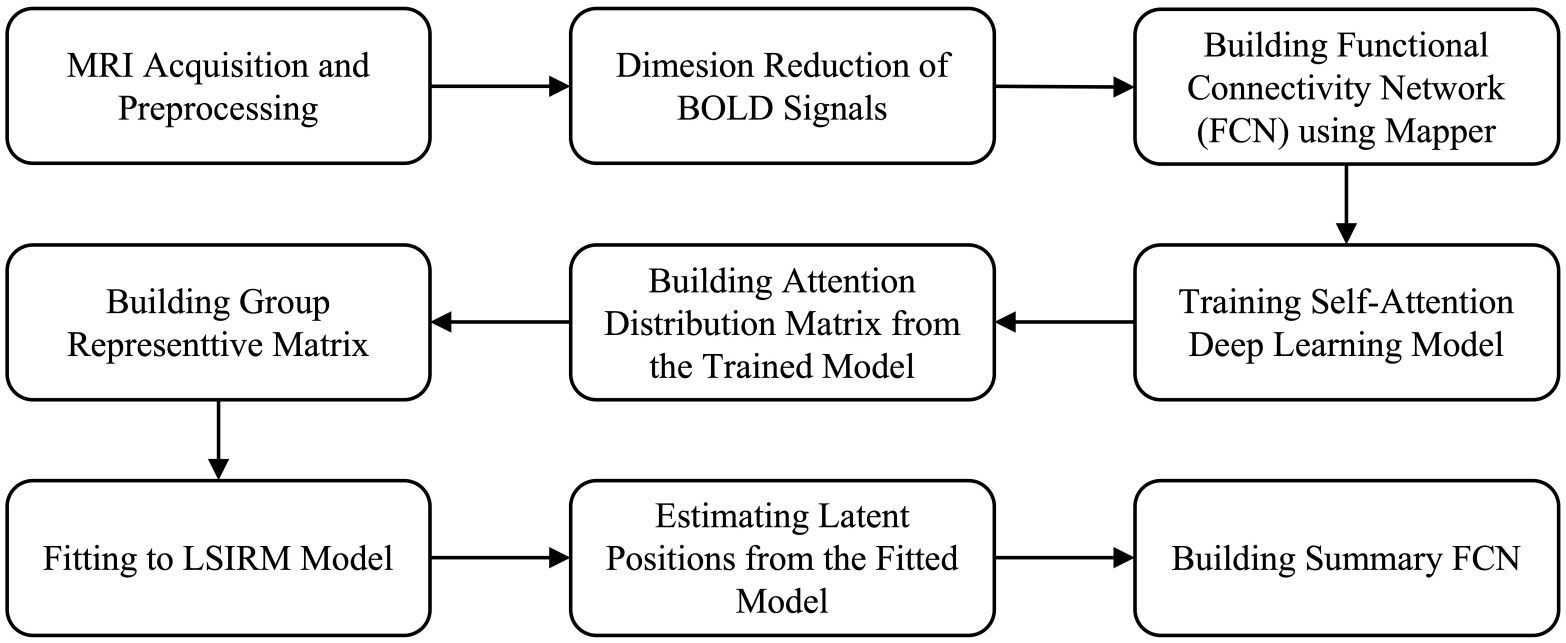
Overview of our fusion analytic framework.

**Figure 2:**
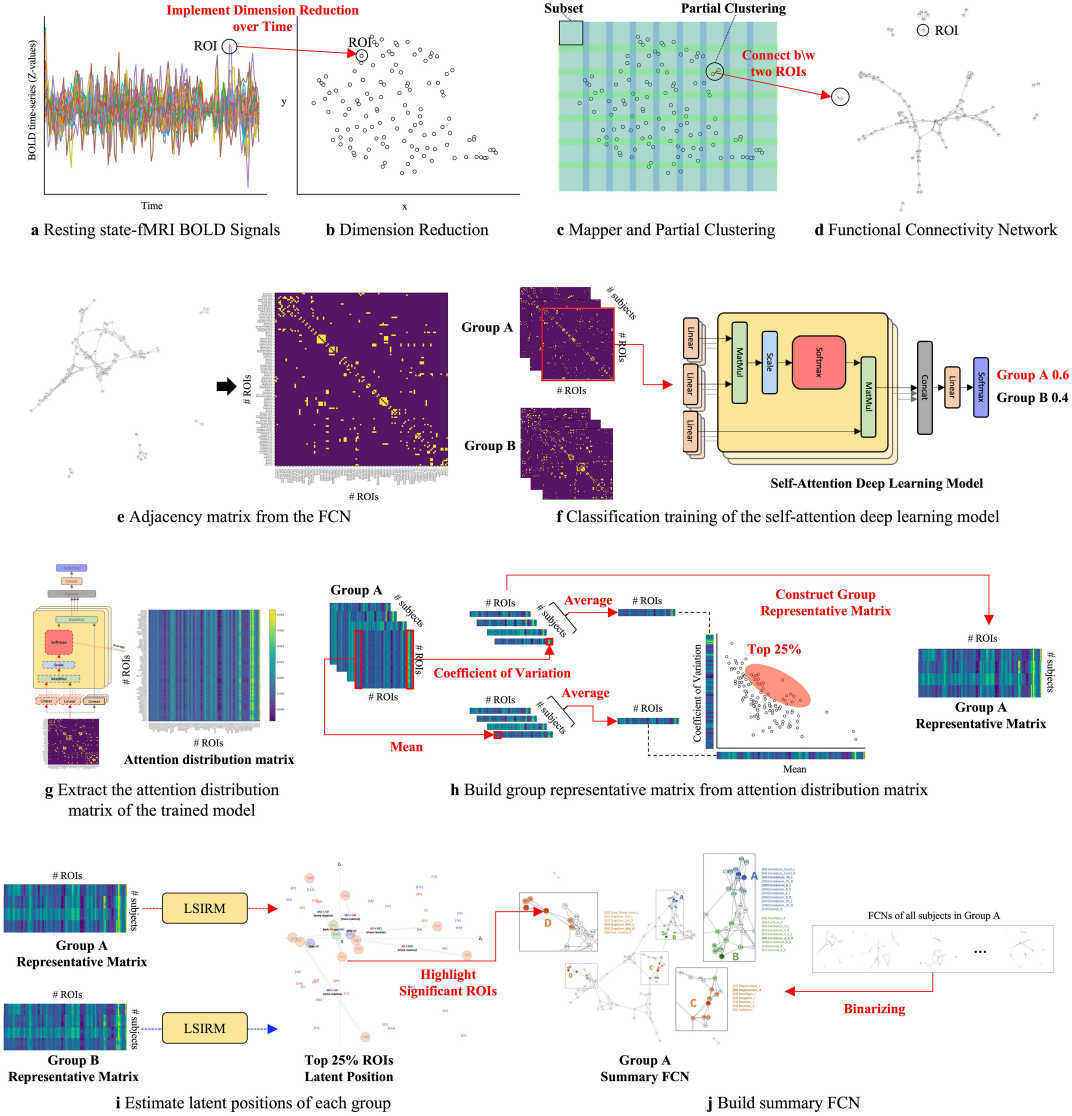
A graphical illustration of the fusion analytic framework with three steps: **Step 1 (A–D)**: constructing the FCN for each group by embedding rs-fMRI BOLD signals into a 2D space using dimensionality reduction; **Step 2 (E–H)**: generating a group representative matrix from the attention distribution matrices; and **Step 3 (I, J)**: identifying meaningful ROIs using LSIRM and marking them on a group summary FCN.

The overview of the proposed framework is illustrated in Figure 1, and detailed aspects are presented in Figure 2. Our framework comprises three key steps. In Step 1 (Figures 2A–D), we construct FCNs for each subject's ROIs by connecting them based on their embedded positions using the mapper algorithm (Chazal and Michel, 2017). To manage the time dimension of rs-fMRI data, we first apply dimensionality reduction, and then use the mapper to construct individual FCNs. While this approach reveals the overall connectivity structure, it remains challenging to identify significant ROI connections that distinguish one group from another. In Step 2 (Figures 2E–H), we perform binary classification using Self-Attn (Vaswani et al., 2017) on the subjects' FCNs and generate an attention distribution matrix for each subject. Next, we use the coefficient of variation (CV) and mean values to build a group-level attention distribution matrix. In Step 3 (Figures 2I–J), we apply LSIRM to the group attention distribution matrix to identify group-representative ROIs. These ROIs are then mapped onto a group summary FCN to extend their connections to other ROIs.

To validate the proposed framework, we applied it to classify rs-fMRI data from different stages of neurodegenerative diseases, which exhibit varying levels of cognitive impairment. We used resting brain scans from the Alzheimer's Disease Neuroimaging Initiative (ADNI) database, which is a multisite longitudinal study widely used for biomarker exploration in Alzheimer's disease diagnosis (Jack Jr et al., 2008; Mueller et al., 2005). We aimed to uncover the distinct features of ROIs between the two groups and compare our results with previous findings from ADNI publications.

## 2 Materials and methods

Our analysis approach involves three main steps: (1) creating an FCN for each subject in each group, (2) estimating an attention distribution matrix using Self-Attn, and (3) extracting group-representative features of ROIs using LSIRM and visualizing them on the group summary FCN. In this study, we applied our analysis framework to identify the specific ROIs that differentiate four comparisons: Alzheimer's Disease (AD) vs. Mild Cognitive Impairment (MCI), AD vs. Early MCI (EMCI), AD vs. Late MCI (LMCI), and EMCI vs. LMCI. We utilized rs-fMRI data collected from AD, EMCI, MCI, and LMCI groups in the ADNI dataset.

### 2.1 ADNI study

The ADNI dataset is composed of four consecutive cohorts (ADNI1, ADNI2, ADNI-GO, and ADNI3). Participants were recruited for initial periods in the ADNI1 cohorts (October 2004). Follow-up of participants were recruited to the ADNI3 cohort period. To facilitate the preprocessing of the brain rs-fMRI data, we selected data with the same MR parameters. Among several MR parameters, three MR parameters were applied as criteria (200 time points, TR = 3000 ms, 48 slices). After filtering based on these three conditions, 281 participants remained in the ADNI2, ADNI-GO, and ADNI3 cohorts. The ADNI1 cohort was excluded because it did not contain data that met the aforementioned conditions. As a result, we used axial rs-fMRI data from 57 AD subjects, 93 EMCI subjects, 53 LMCI subjects, and 78 MCI subjects (Table 1, Figure 2A). By focusing on these specific disease comparisons, we aim to uncover the key ROIs that exhibit distinct patterns and contribute significantly to the classification and differentiation of these cognitive impairment conditions. All data are publicly available, at http://adni.loni.usc.edu/.

**Table 1:** Demographic and clinical information (mean ± standard deviation) of the studied ADNI subjects.

	**Female%**	**Age (mean±SD)**	**MMSE (mean±SD)**	**Global CDR (mean±SD)**
AD	38.6%	74.39 ± 8.26	21.03 ± 4.08	0.98 ± 0.51
EMCI	47.3%	75.78 ± 6.90	28.70 ± 1.64	0.09 ± 0.36
LMCI	35.8%	75.03 ± 7.29	26.55 ± 3.46	0.58 ± 0.34
MCI	48.7%	74.81 ± 8.71	27.20 ± 2.25	0.47 ± 0.23

### 2.2 MRI acquisition

The participants included in this study participated in scanning at diverse sites using 3T MRI scanners manufactured by Philips Medical Systems and Siemens Healthineers. The detailed MRI protocols of the ADNI dataset were reported on the webpage (http://adni.loni.usc.edu/methods/mri-tool/mri-acquisition/). In the ADNI2 and ADNI-GO cohorts, MRI scanning was performed at 26 different sites with Philips 3T MRI scanners, using synchronized scanning parameters. In the case of the ADNI3 cohort, Siemens 3T MRI scanners were used to collect fMRI data with synchronized parameters.

### 2.3 MRI preprocessing

The rs-fMRI datasets, originally formatted in Digital Imaging and Communications in Medicine (DICM), were converted to the Neuroimaging Informatics Technology Initiative (NITI) format, the standard in fMRI research. This conversion preserved all original slices across four dimensions (x, y, z, and time). The preprocessing steps began with slice timing correction to account for the acquisition order of slices, as required by the MRI protocol. Since all rs-fMRI data were acquired using interleaved scanning (an alternating acquisition method), the slice order was applied accordingly, alternating between even- and odd-numbered slices (1 to 48). Following slice timing correction, realignment and head motion correction were performed to address misalignment or movement artifacts.

Next, the corrected rs-fMRI data were spatially normalized to a 3 mm isotropic voxel size using an EPI template, ensuring anatomical consistency across brain MR images. Smoothing was applied using a Gaussian kernel (FWHM = 6 mm, full-width at half-maximum) to optimize spatial resolution and reduce noise. To account for scanner drift and physiological fluctuations, linear trends were removed, and covariates such as white matter (WM) and cerebrospinal fluid (CSF) signals were regressed out, isolating relevant brain activation data. Temporal bandpass filtering (0.01–0.1 Hz) was then applied to minimize low- and high-frequency noise, preserving fluctuations related to intrinsic brain activity.

Finally, the Automatic Anatomical Labeling (AAL) atlas, which segments the brain into 116 regions, was used to define brain regions and extract ROI time-course data. All preprocessing steps were conducted using the Data Processing and Analysis of Brain Imaging toolbox (DPABI, Version 5.3, available at http://rfmri.org/dpabi), and Statistical Parametric Mapping (SPM, Version 12, available at www.fil.ion.ucl.ac.uk/spm/software/spm12/), both implemented in MATLAB R2020b (MathWorks, Natick, MA, USA) (Yan et al., 2016).

### 2.4 Functional connectivity networks

Dimension reduction methods are well-established techniques for embedding complex and structured data, including Principal Component Analysis (PCA) (Dunteman, 1989), T-distributed Stochastic Neighbor Embedding (t-SNE) (van der Maaten and Hinton, 2008), and Uniform Manifold Approximation and Projection (UMAP) (McInnes et al., 2018). Both t-SNE and UMAP model the manifold using stochastic and topological information. Respectively, t-SNE converts neighborhood distances into conditional probabilities that represent similarity, while UMAP employs a fuzzy simplicial complex with edge weights that reflect the likelihood of connectivity.

#### 2.4.1 Dimension reduction

PCA (Dunteman, 1989) is a technique that uses an orthogonal transformation to reduce high-dimensional data to low-dimensional data (main components) that are not linearly related. The axis with the most significant variance is the first principal component, and the second greatest variance is the second principal component. This decomposition divides the sample into components that best represent the differences. On the other hand, t-SNE (van der Maaten and Hinton, 2008) is a non-linear dimension reduction method that aids in understanding the data with impact information. It is based on t-distribution, which has a heavier tail than normal distribution that helps cover up the far distribution element of high-dimensional data. The t-SNE results depict the embedded points whose distances, trained by calculating the points' similarity in structure, reflect their degree of similarity. UMAP (McInnes et al., 2018) is a nonlinear dimension reduction method that models the manifold using a topological structure. Because it is based on topological space, the embedding points are close in proximity if the two data points have similar topological features. It first reorganizes the data into a fuzzy simplicial complex. This complex produces the connections based on the hyper-parameter that controls the connectivity around the data. Then, it projects the correlated structured data into a low-dimensional space based on their connection, where the connection indicates the proximity.

#### 2.4.2 Mapper

Mapper is one of the techniques derived from topological data analysis, which enables the representation of the topological structure of high-dimensional data as a network. Topological data analysis simplifies the complexity of the topological space by transforming it into a network consisting of nodes and connections that capture the topological characteristics, such as points, lines, and triangles. The Mapper process involves two main steps. First, the high-dimensional topological space is mapped onto a real-valued measure space represented as a graph. This mapping function can be any real-valued function that captures the essential features of the data. For example, dimensionality reduction techniques like PCA, t-SNE, and UMAP use real-valued functions to project high-dimensional data into a lower-dimensional space such as Euclidean space. In the next step, the mapper partitions the graph into subsets of data, and each subset is clustered to define connections. This process identifies the structural relationships within the data. This process is called the *Mapper*, where each sub-cluster is treated as a node, and nodes are connected when they share similar data attributes.

The Mapper can be considered a form of partial clustering. It applies a standard clustering algorithm to subsets of the original data and examines the interactions between the resulting sub-clusters. When two non-empty subsets U and V are considered, their sub-clusters may have overlapping elements that construct a simplicial complex. The sub-clusters are referred to as vertices (or nodes), while the overlapping elements form edges in the complex. This process yields a simplicial complex consisting of points, lines, and triangles, which provides insights into the topological structure of high-dimensional data.

### 2.5 Attention distribution matrix

The attention distribution matrix is obtained from Self-Attn, representing a probability distribution that captures the relationships between input elements that play a key role in performing a task. The training phase, as detailed in Algorithm 1, is shown in Figure 2F, where the FCN is trained using Self-Attn. In this phase, the model learns the relationships between ROIs from the input FCN and identifies which network of ROIs characterizes the unique features of a given group. The inference phase corresponds to Figure 2G, where the trained model takes the FCN of a subject as input and extracts values from a specific layer, generating the attention distribution matrix. This layer captures the relationships between ROIs in parallel, in the form of a probability distribution, thus capturing diverse interaction patterns among the ROIs. Finally, these relationships are averaged to produce a single matrix which is called the attention distribution matrix. This matrix explains the relationships between ROIs that contribute to classifying a subject into a specific group.

**Algorithm 1:** Self-Attn for training attention distribution matrix.

**Require:** Data $D=\{{\left(FCN^{\left(i\right)},group^{\left(i\right)}\right\}}_{i=1}^{N}$, where *group*^(*i*)^ is the group label for each subject *i*, *FCN*^(*i*)^∈ℝ^116 × 116^, number of attention heads *H* = 128
**Ensure:** Attention distribution matrix set ${\left\{attnM^{\left(i\right)}\right\}}_{i=1}^{N}$, where *attnM*^(*i*)^∈ℝ^116 × 116^
1: Split D into training and test datasets
2: Initialize Self-Attn with *H* heads
3: Train Self-Attn using the training data ${\left\{\left(FCN^{\left(i\right)},group^{\left(i\right)}\right)\right\}}_{i=1}^{N_{train}}$
4: **After training:**
5: **for** each subject *i* = 1 to *N* **do**
6: Feed *FCN*^(*i*)^ into the trained Self-Attn
7: Extract attention weights $attn_{h}^{\left(i\right)}\in {\mathbb{R}}^{116\times 116}$ for each attention head *h* = 1, 2, …, *H*
8: Compute the Attention Distribution Matrix $attnM^{\left(i\right)}=\frac{1}{H}\overset{H}{\underset{h=1}{\sum }}attn_{h}^{\left(i\right)}$, where *attnM*^(*i*)^∈ℝ^116 × 116^
9: **end for**
10: Collect all Attention Distribution Matrices: ${\left\{attnM^{\left(i\right)}\right\}}_{i=1}^{N}$
11: **return** ${\left\{attnM^{\left(i\right)}\right\}}_{i=1}^{N}$

In particular, Self-Attn with multi-head self-attention can distinguish between groups but also explains the relationships among ROIs that contribute to group classification. Multi-head self-attention is one of the core mechanisms of the Transformer model. It is highly effective in learning how different elements in input data are related to each other (Vaswani et al., 2017). It is particularly useful for exploring interactions between brain regions (Zhao et al., 2022, 2024). Self-attention evaluates each element's relationships, focusing on the more important elements based on this information. This is particularly useful for capturing the interactions between ROIs and identifying relationships that distinguish groups (Velickovic et al., 2017; Lei et al., 2022; Zhang et al., 2022).

Key components of the multi-head self-attention mechanism, which plays an important role in executing self-attention, are known as Query (**Q**), Key (**K**), and Value (**V**). These components are used to learn the relationships between input elements. **Q** functions as a ‘query,' capturing how a specific ROI relates to other ROIs. **K** holds the features of each ROI, encapsulating the information each ROI possesses, and is used together with **Q** to assess the relevance between ROIs. For instance, if the dot product between **Q** and **K** is high, the corresponding ROIs have highly significant interactions. **V** contains the actual information of each ROI and is propagated using the attention weights derived from **Q** and **K**. For example, if a particular ROI receives high attention weights, it extracts crucial information from the **V** of other ROIs, generating more meaningful outputs.

The input consists of **Q**, **K**, and, **V**, all having a dimensionality of *d*_*k*_. The computation involves calculating the dot products between **Q** and each **K**, dividing the result of each by the square root of *d*_*k*_, and then applying a softmax function to obtain weights corresponding to **V**. If we consider a total of **R** ROIs, we can represent **Q**, **K**, and **V** as element of ${\mathbb{R}}^{\text{R}\times d_{k}}$. By applying softmax to the attention scores obtained from the dot product of **Q** and **K**, we derive the attention weights, denoted by attn(**Q**, **K**). These attention weights represent the relationships between ROIs and indicate the probability distribution of how much each ROI should focus on other ROIs. The final output is obtained by multiplying **V** with attn(**Q**, **K**).


(1) $$\frac{attn(Q,K)=\text{Softmax}(\frac{QK^{⊤}}{\sqrt{d_{k}}})}{\text{Attention}(Q,K,V)=attn(Q,K)V}$$

Equation 1 is extended into multi-head self-attention as shown in Equation 2 to capture various aspects of ROIs, particularly in high-dimensional datasets. Here, with *H* heads, *head*_*h*_ where *h* = 1, ⋯ , *H*, each *head*_*h*_ layer consists of weight matrices $W_{h}^{Q}$, $W_{h}^{K}$, and $W_{h}^{V}$, which applied to the output layer. The multi-head self-attention is formed by concatenating these *H* sets of *head*_*h*_. The resulting output is passed through a multi-layer perceptron.


(2) $$\begin{array}{c} \text{MultiHead}\left(\text{Q},\text{K},\text{V}\right)=\text{Concat}\left({\text{head}}_{1},...,{\text{head}}_{H}\right)W^{O}\\ \text{where}\;head_{h}=\text{Attention}\left(\text{Q}W_{h}^{\text{Q}},\text{K}W_{h}^{\text{K}},\text{V}W_{h}^{\text{V}}\right) \end{array}$$

In the end, we obtain the attention distribution matrix from the trained model via the inference phase (Figure 2G). For each input, the attention distribution matrix *attnM*(**Q**, **K**) is obtained by averaging the attention layers across all *H* heads, denoted as *attn*_*h*_.


(3) $$\begin{array}{c} attnM\left(\text{Q},\text{K}\right)=\frac{\sum_{i=1}^{H}attn_{h}}{H}\\ \text{where}attn_{h}=attn\left(\text{Q}W_{i}^{\text{Q}},\text{K}W_{i}^{\text{K}}\right) \end{array}$$

### 2.6 Group representative ROIs features using Latent Space Item-Response Model

LSIRM (Jeon M. et al., 2021) is a model that represents item-response datasets as bipartite networks, estimating interactions between items (ROIs) and respondents (subjects). In our study, we aim to estimate the latent positions of ROIs based on the interactions between subjects and ROIs. Here, the “Interaction” is measured by the degree of value between subjects and ROIs, indicating the association between subjects and ROIs. These patterns are visualized by estimating the latent positions in space, which can be in Euclidean space, allowing for a more intuitive understanding of these associations. The original LSIRM model is designed for a binary item-response data (0 or 1) (Embretson and Reise, 2013). Therefore, we adopt a continuous version of LSIRM to apply it to group representative matrix **X**_*h*|*g, h*_. Each cell value *y*_*ij*_ represents the coefficient of variation of ROI *j* in the attention distribution of subject *i* from group *h* compared to group *g*. This is continuous for *i* = 1, ⋯ , *N*_*h*_, and *j* = 1, ⋯ , *R*. Equation 4 shows the continuous version of LSIRM:


(4) $$\begin{array}{c} \mathbb{P}\left(y_{ij}|\Theta \right)\sim \text{Normal}\left(\theta_{j}+\beta_{i}-||{\text{u}}_{j}-{\text{v}}_{i}||,\sigma^{2}\right), \end{array}$$

where **Θ** represents {**θ** = {θ_*j*_}, **β** = {β_*i*_}, $\text{U}={\left\{{\text{u}}_{j}\right\}}_{j=1}^{R}$, $\text{V}={\left\{{\text{v}}_{i}\right\}}_{i=1}^{N_{h}}$} and ||**u**_*j*_−**v**_*i*_|| denotes the Euclidean distance between subject *i* and ROI *j*. LSIRM consists of two parts, the attribute part and the interaction part. In the attribute part, there are two parameters: θ_*j*_∈ℝ and β_*i*_∈ℝ. The parameter β_*i*_ represents the degree of responses for the subject *i*, while θ_*j*_ represents the responses for ROI *j*. In the interaction part, we have the latent configurations **u**_*j*_ and **v**_*i*_ for each ROI *j* and subject *i*, respectively.

If subject *i* shows a high value in ROI *j*, the corresponding association is relatively strong, which is represented through their distance **u**_*j*_−**v**_*i*_. Therefore, their latent positions **u**_*j*_ and **v**_*i*_ become closer because of a smaller distance compared to other associations. Conversely, if subject *i* shows low values in ROI *j*, the association is weaker, and the distance between their latent positions **u**_*j*_ and **v**_*i*_ is comparably larger. Based on the latent positions of ROIs and subjects, we can interpret the overall relationships that are inherent in data. Algorithm 2 outlines the detailed training procedure for LSIRM.

**Algorithm 2:** LSIRM training process using Markov chain Monte Carlo (MCMC).

**Require:** Data ${\text{X}}_{h|g,h}={\left\{{\left\{y_{ij}\right\}}_{i=1}^{N_{h}}\right\}}_{j=1}^{R}$ and hyperparameters *a* and *b* for priors $\sigma_{\theta }^{2}$
**Ensure:** Posterior samples of latent positions **u** (ROIs), **v** (subjects), **β** (ROI difficulties), and **θ** (subject abilities)
1: Initialize **u**, **v**, **β**, **θ** randomly
2: **for** each MCMC iteration **do**
3: **for** each subject *i* = 1 to *N*_*h*_ **do**
4: Sample θ_*i*_ from its full conditional posterior:
$\theta_{i}^{*}\sim \text{Normal}\left(0,\sigma_{\theta }^{2}\right)$
where $\sigma_{\theta }^{2}$ are based on the likelihood and prior.
5: Compute the acceptance probability for θ_*i*_:
$\alpha_{\theta_{i}}=\min\left(1,\frac{P\left(\overset{*}{\underset{i}{\theta }}|\text{y},\sigma_{\theta }^{2},\beta ,\text{u},\text{v}\right)}{P\left(\theta_{i}|\text{y},\sigma_{\theta }^{2},\beta ,\text{u},\text{v}\right)}\right)$
6: Accept or reject $\theta_{i}^{*}$.
7: **end for**
8: Sample subject-specific variance $\sigma_{\theta }^{2}$ from its full conditional posterior:
$\sigma_{\theta }^{2}\sim \text{Inv-Gamma}\left(a+\frac{N}{2},b+\frac{1}{2}\overset{N}{\underset{i=1}{\sum }}\theta_{i}^{2}\right)$
9: **for** each ROI *j* = 1 to *R* **do**
10: Sample β_*j*_ from its full conditional posterior:
$\beta_{j}^{*}\sim \text{Normal}\left(0,1\right)$
11: Compute the acceptance probability for β_*j*_:
$\alpha_{\beta_{j}}=\min\left(1,\frac{P\left(\overset{*}{\underset{j}{\beta }}|\text{y},\theta ,\text{u},\text{v}\right)}{P\left(\beta_{j}|\text{y},\theta ,\text{u},\text{v}\right)}\right)$
12: Accept or reject $\beta_{j}^{*}$.
13: **end for**
14: **for** each subject *i* = 1 to *N*_*h*_ **do**
15: Sample latent position **v**_*i*_ from its full conditional posterior:
${\text{v}}_{i}^{*}\sim \text{Normal}\left(0,1\right)$
16: Accept or reject ${\text{v}}_{i}^{*}$ based on its acceptance probability.
17: **end for**
18: **for** each ROI *j* = 1 to *R* **do**
19: Sample latent position **u**_*j*_ from its full conditional posterior:
${\text{u}}_{j}^{*}\sim \text{Normal}\left(0,1\right)$
20: Accept or reject ${\text{u}}_{j}^{*}$ based on its acceptance probability.
21: **end for**
22: **end for**
23: Return posterior samples for **u**, **v**, **β**, **θ**, $\sigma_{\theta }^{2}$

Additionally, we can understand the significance of the latent positions of ROIs. When the latent positions of ROIs are located near the center, it indicates that most subjects respond similarly to these ROIs. This is because, when estimating the latent positions of these ROIs, the positions of all subjects are considered simultaneously, resulting in a minimization of the distance between the latent positions. Consequently, the latent positions of commonly reacted ROIs are near the center, allowing most subjects to exhibit similar reactions. This property facilitates the extraction of commonly reacted ROIs for each group *h* compared to group *g*, enabling the construction of the group representative matrix **X**_*h*|*g, h*_.

### 2.7 Advantages of Self-Attn and LSIRM in capturing fMRI intricacies

Self-attention (Vaswani et al., 2017) evaluates the relationships between elements in the input sequence, and assigns weights to emphasize important information from these relationships. This mechanism enables the model to consider all interactions between ROIs in a single computation. Furthermore, because it processes all ROIs simultaneously, it is useful for capturing long-range dependencies which helps capture global information. Multi-head self-attention enables learning in parallel interactions between ROIs, allowing the model to capture diverse relationships and patterns in greater depth.

In contrast, eXtreme Gradient Boosting (XGBoost) (Chen and Guestrin, 2016) is an ensemble method based on decision trees that is focused on identifying classification rules between each data element and the target, instead of considering the relationships among elements. In other words, XGBoost classifies based on specific ROI values. However, when data patterns are unclear, such as in rs-fMRI, it is also important to understand the overall network of ROI relationships. XGBoost can have difficulty capturing these complex underlying interactions (Mørup et al., 2010; Mart́ınez-Riaño et al., 2023).

On the other hand, Multi-Layer Perceptron (MLP) (Qiu et al., 2020) primarily analyzes global features instead of local patterns, leading to the loss of information about the interactions between specific ROI features. MLP processes all input data features simultaneously, and as data passes through each layer, it gets transformed using non-linear activation functions. However, this process does not learn the interactions between the inputs, since all inputs are equally processed throughout the network. While MLP can easily capture global characteristics, they have limitations in training complex interactions between ROIs (Lai and Zhang, 2023).

On the other hand, Convolutional Neural Networks (CNN) (Zunair et al., 2020) extract features by sliding small filters over the data. However, it is difficult for CNN to capture global relationships within the data through a single convolution operation. To address this, multiple layers are required, which necessitates more data and increases the training time needed to learn global relationships. While CNN is effective at learning local interactions between adjacent ROIs, they have limitations in capturing the global interactions between ROIs (Wang et al., 2018).

For these reasons, Self-Attn is particularly well-suited for learning interactions between ROIs, as it can simultaneously capture local information and global patterns. This capability makes Self-Attn effective at capturing the complex signals within rs-fMRI data. As shown in Table 2, this results in superior performance compared to other models.

**Table 2:** Performance comparison of classification accuracy using 10-fold cross-validation between Self-Attn, two previous studies (Liu et al., 2020; Wee et al., 2019), and three models [XGBoost (Chen and Guestrin, 2016; Qiu et al., 2020), and CNN (Zunair et al., 2020)].

**Method**		**AD/MCI**	**EMCI/AD**	**LMCI/AD**	**EMCI/ LMCI**
Liu et al.		0.8890	-	-	-
Wee et al.		-	0.7920	0.6520	0.6090
XGBoost	Pearson	0.7949	0.8206	0.8511	0.7802
	Fisher	0.7460	0.6254	0.6353	0.6776
MLP	Pearson	0.8132	0.7600	0.7809	0.7457
	Fisher	0.7835	0.7533	0.7900	0.7605
	Linear	0.7461	0.7333	0.7146	0.7452
	Stochastic	0.7659	0.7600	0.6427	0.7381
	Topological	0.7819	0.7067	0.6518	0.6838
CNN	Pearson	0.8654	0.7733	0.7355	0.7667
	Fisher	0.8648	0.7390	0.7809	0.7267
	Linear	0.7747	0.7586	0.7891	0.7600
	Stochastic	0.8176	0.7657	0.7246	0.8200
	Topological	0.8192	0.7600	0.7155	0.7600
Self-Attn	Pearson	0.8659	0.8067	0.8809	0.8071
	Fisher	0.8813	0.8133	0.8718	0.8152
	Linear	0.9022	0.8467	0.8627	0.8624
	Stochastic	0.9033	**0.8867**	0.8900	**0.8971**
	Topological	**0.9104**	0.8733	**0.9173**	0.8695

The bold values indicate the models with the highest accuracy in each comparison, which were selected as the optimal models for subsequent analysis.

Our approach uses LSIRM to visualize the interactions among ROIs, reflected in the group representative matrix derived from the attention distribution matrix. LSIRM captures these interactions by estimating the latent positions of ROIs, providing an intuitive interpretation of their relationships. Importantly, the group representative matrix is not an adjacency matrix, but a subject-by-ROI matrix, where the goal is to identify group-level features rather than individual-specific ones. Since LSIRM considers all subject-ROI interactions, it identifies common ROI features that show consistent reactions across all subjects. Therefore, by employing LSIRM, our approach can achieve a better understanding of group effects and provide a more intuitive way to interpret ROI interactions.

## 3 Results

In this study, we applied our analysis framework to identify the specific ROIs that differentiate between four disease comparisons: AD vs. MCI, AD vs. EMCI, AD vs. LMCI, and EMCI vs. LMCI. We utilized rs-fMRI data collected from AD, EMCI, MCI, and LMCI from the ADNI dataset.

### 3.1 Step 1: functional connectivity networks of each group

First, we constructed an FCN among brain regions based on their rs-fMRI Blood-Oxygen-Level-Dependent (BOLD) signals. We used AAL-116 templates to extract 116 rs-fMRI BOLD signals, representing different brain regions. Supplementary Table S1 in supporting information contains detailed information about the AAL-116 templates. Due to the high-dimensional and correlation structure of fMRI data, we implemented dimension reduction such as PCA, t-SNE, and UMAP over time to embed the high-dimensional correlated structure dataset into low two-dimensional space (Figure 2B). We empirically searched for the optimal combination of UMAP hyperparameters to enhance prediction accuracy. Based on this, we chose the number of neighbors to be 15 and set the minimum distance to 0.1, consistent with hyperparameters used in previous studies for visualizing high-dimensional data, such as genomics (Becht et al., 2019) and single-cell data (Diaz-Papkovich et al., 2021).

To mitigate the subjectivity in determining relevance among ROIs, we employed Mapper (Chazal and Michel, 2017), a partial clustering method, to identify significant connections between ROIs (represented as Figure 2C). ROIs assigned to the same cluster were considered connected. Subsequently, we generated FCNs for each set of embedded ROIs from different dimension reduction methods (represented as Figure 2D). These FCNs captured relationships and connectivity patterns within the high-dimensional correlated fMRI data, representing the data as a connectivity network.

**Figure 3:**
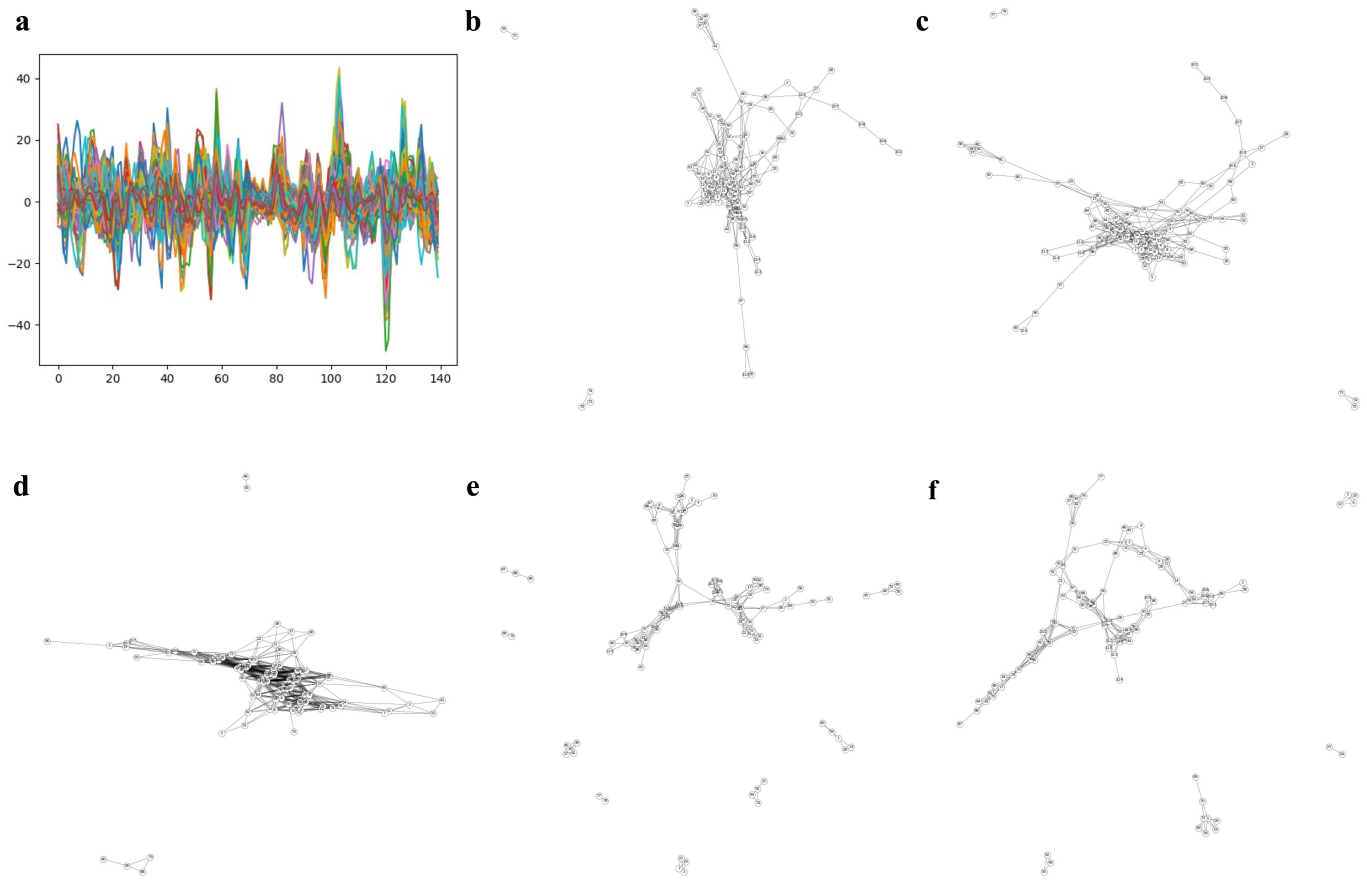
Correlation- and dimension reduction-based FCNs for an AD subject. **(A)** shows ROIs and rs-fMRI BOLD signals for an AD subject. FCNs were generated using correlation-based methods: Pearson's r and Fisher's z (shown in **B, C**). To better understand the interrelationships between brain regions, dimension reduction techniques were applied to estimate latent positions of ROIs: PCA in **D** (linear space), t-SNE in **E** (stochastic space), and UMAP in **F** (topological space).

Figure 3 and Supplementary Figures S1–S3 show each subject's rs-fMRI BOLD signals and two types of FCNs: correlation- and dimension-reduction-based FCNs obtained from dimension reduction methods corresponding to AD, MCI, EMCI, and LMCI. Through correlation- and dimension-reduction-based approaches, those different perspectives enabled a comprehensive understanding of their structural characteristics. Note that these FCNs are input for Self-Attn to classify between two diseases. One can apply other dimensionality reduction methods to obtain the embedded ROIs; however, one can select the optimal method based on the prediction accuracy provided by the Self-Attn, where the input is FCNs.

### 3.2 Step 2: attention distribution matrix from self-attention deep learning model

We focus on identifying which ROIs are important features for distinguishing groups in terms of ROIs' interactions with others. In the previous step, we applied dimension reduction techniques such as PCA, t-SNE, and UMAP to the original time-series of the ROIs to construct FCNs. Additionally, we compared these findings with two correlation matrices calculated from the original time-series data of the ROIs: (1) Pearson's r and (2) Fisher's z, both of which capture the associations among ROIs. With this data, we employed Self-Attn, using FCNs or correlation matrices as input, with the target being a binary indicator of group membership.

In the Transformer architecture, *d*_*k*_ is typically defined by dividing the total input dimension (*d*_*model*_) by the number of attention heads (*h*) (Vaswani et al., 2017). However, the roles of the various attention heads vary, and not all heads contribute equally to model performance. Therefore, focusing on the most important heads can maintain model performance while reducing the risk of overfitting (Voita et al., 2019; Michel et al., 2019). To optimize classification accuracy while avoiding overfitting and underfitting, we conducted a parameter search for *h* = {16, 32, 64, 128}. By modifying *h*, we implicitly explored the corresponding *d*_*k*_ as well, specifically investigating *d*_*k*_ = {2, 4, 8} where setting *d*_*k*_ to a small value is practically effective specifically in self-attention (Tay et al., 2022). We validated the model through a 10-fold cross-validation, and showed that the configuration with *h* = 128 and its corresponding *d*_*k*_ = 2 achieved consistently robust classification performance. We provide details for parameter search in Supplementary Figure S4. We applied the model to 116 ROIs using the following settings: the batch size of 8, a dropout rate of 0.9, Adam optimizer (Kingma and Ba, 2014), the learning rate of 0.01, and cross-entropy loss.

Table 2 shows the performance of Self-Attn. We compared the classification performance against recent studies (Liu et al., 2020; Wee et al., 2019) and baseline models [XGBoost (Chen and Guestrin, 2016), MLP (Qiu et al., 2020), and CNN (Zunair et al., 2020)]. Our method outperforms all other approaches across the disease group comparisons. Notably, the stochastic and topological-based FCN, which captures hidden connectivity among ROIs, achieved the highest accuracy.

Using Self-Attn, we obtained the attention distribution (Figure 2F) by each *i*th subject from group *g*. These attention distributions, denoted as ${\text{A}}_{\left(q,r\right)}^{\left(i\right)}\in {\mathbb{R}}^{116\times 116}$, where *i* = 1, ⋯ , *N*_*g*_. Here, *N*_*g*_ indicates the number of subjects from each disease group *g* = {*AD, MCI, EMCI, LMCI*}, where *N*_AD_ = 57, *N*_MCI_ = 78, *N*_EMCI_ = 93, and *N*_LMCI_ = 53. These attention distributions ${\text{A}}_{\left(q,r\right)}^{\left(i\right)}$ reveal the features that the model focused on when classifying subjects in each disease group against the other comparison groups.

We considered this attention distribution ${\text{A}}_{\left(q,r\right)}^{\left(i\right)}$ as the matrix ${\text{Y}}_{g|g,h}^{\left(i\right)}$, for *g*≠*h* and *g, h* = 1, ⋯ , *G*, where each row and column corresponds to ROIs, and the values indicate the significance of each ROI's contribution to the classify the subject *i* in group *g* against group *h* (Vig, 2019) (Figure 2G). Although the resting-state data shows low signal levels, the classification accuracy of 90% demonstrates that the attention matrices effectively distinguish between the two disease group comparisons.

**Figure 4:**
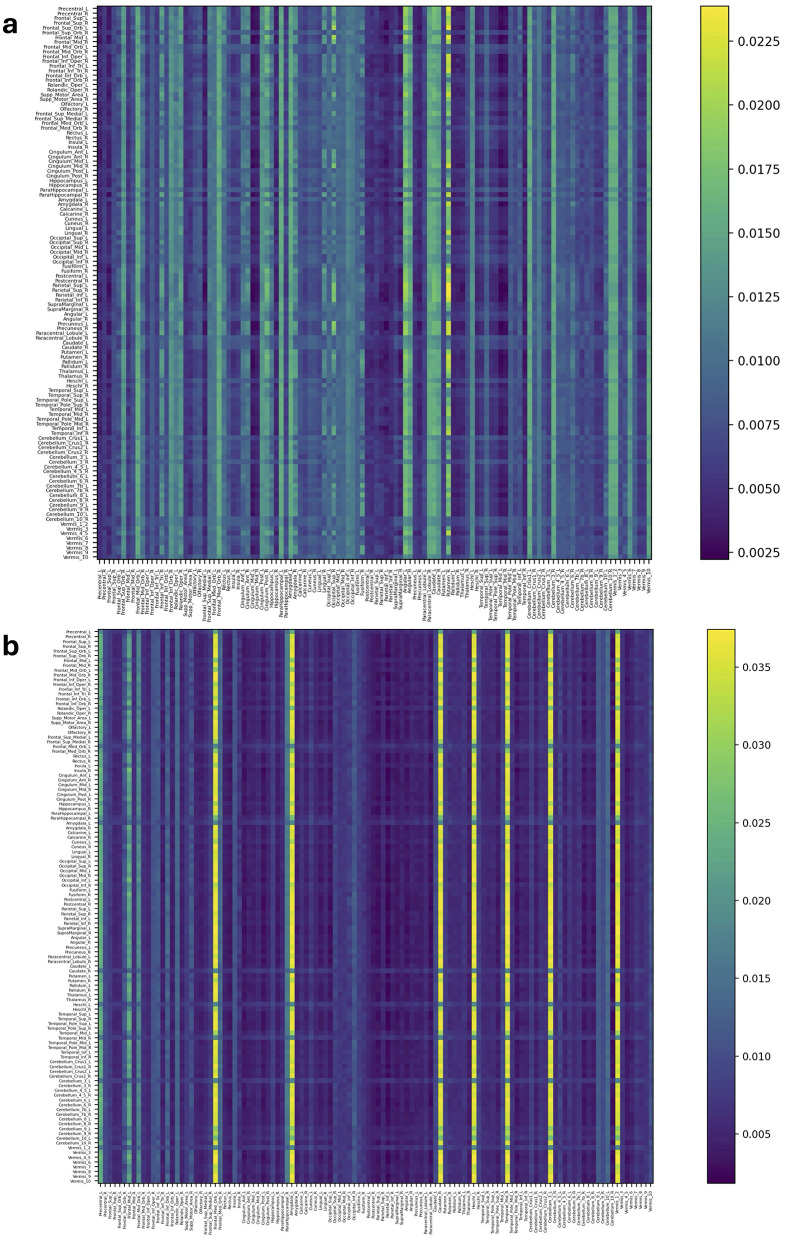
Attention distribution matrix of two subjects within the AD group **(A, B)**, generated by Self-Attn designed for AD and MCI classification, utilizing topological-based FCNs as input.

**Figure 5:**
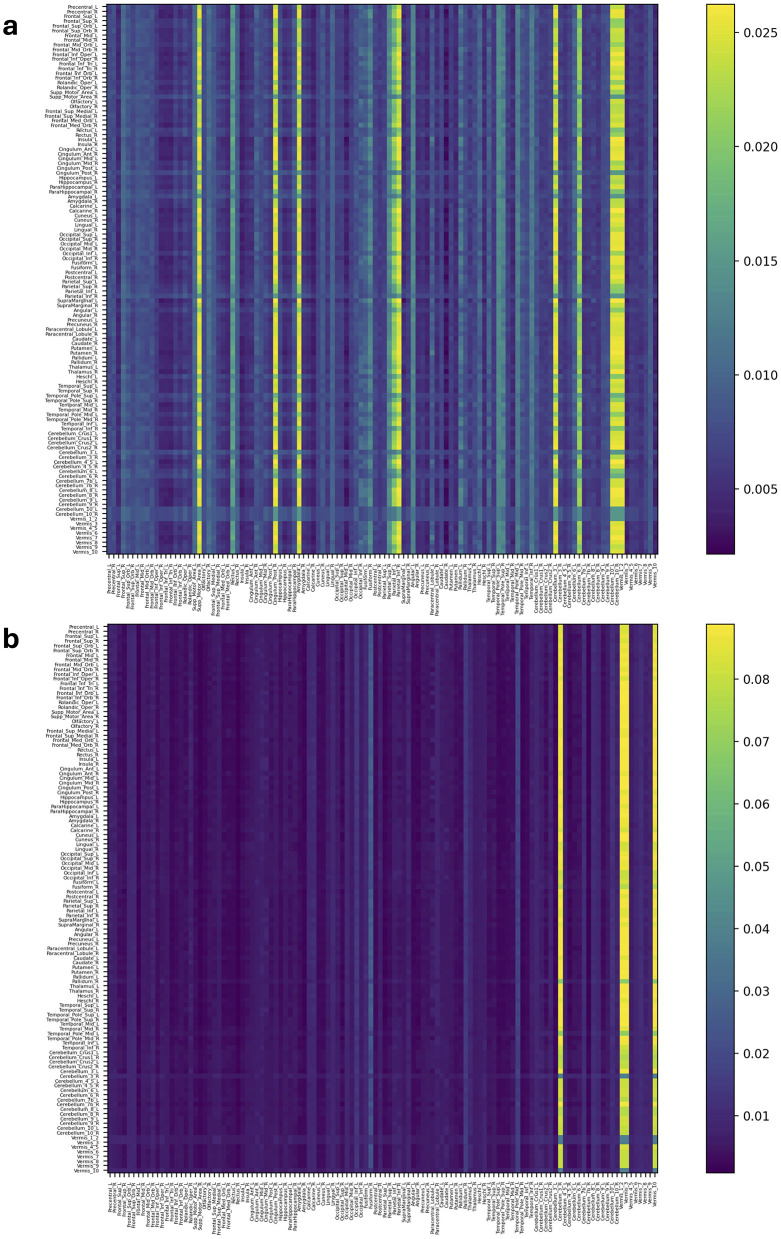
Attention distribution matrix of two subjects within the MCI group **(A, B)**, generated by Self-Attn designed for AD and MCI classification, utilizing topological-based FCNs as input.

Figures 4, 5 and Supplementary Figures S5, S6 represent attention matrices for four randomly selected subjects from the AD and MCI groups, respectively. These matrices are the outcomes of Self-Attn employed for AD and MCI classification, utilizing FCNs derived from topological dimension reduction techniques as inputs. ROIs with Higher attention values suggest their significance in classifying subjects. For instance, in Figure 4A, Putamen_R shows a higher attention value, which is a consistent result in Putamen's volume in the AD group (de Jong et al., 2008). Furthermore, Angular and Paracentral areas have high values indicating that Self-Attn focuses on these regions to assign this subject to the AD category over MCI. Similarly, in Figure 4B, Supplementary Figure S5A, Caudate_R, Caudate_L, ParaHippocampal_L, Cerebellum, Cingulum_Ant_L, and Cingulum_Ant_R have high values which also show significant ROIs marker in AD group (Kesslak et al., 1991; Bobinski et al., 1999; He et al., 2007; Catheline et al., 2010).

On the other hand, Figure 5A has a high value in Supp_Motor_Area_R and Cingulum_Post_R (Lin et al., 2014) which ROIs linked to motor function and exercise (Schmahmann et al., 2001; Aggarwal et al., 2006; Bai et al., 2011). Similarly, in Figure 5B and Supplementary Figures S5A–S6B, Cerebellum, Vermis (van de Mortel et al., 2021), Thalamus_R (Li et al., 2013; Cai et al., 2015), Hippocampus_L, and ParaHippocampal_R (Hämäläinen et al., 2007) emerge as significant ROIs in the MCI group. These patterns in attention distribution explain why Self-Attn places greater emphasis on classifying this subject within the MCI category rather than AD.

Based on these self-attention distributions, the classification accuracy outlined in Table 2  highlights the effectiveness of our well-trained Self-Attn. Nonetheless, individually interpreting each subject within each group can be time-consuming, and there may be potential noise originating from individual differences within each attention distribution matrix.

To identify the representative features of ROIs connections that differentiate between two groups (e.g., *g* and *h*), we constructed a group representative matrix ${\text{X}}_{g|g,h}\in {\mathbb{R}}^{N_{g}\times 116}$ from each attention distribution matrix ${\text{Y}}_{g|g,h}^{\left(i\right)}$ for *i* = 1, ⋯ , *N*_*g*_, where each row represents a subject and each column represents an ROI (Figure 2H). The values in the *g*th group representative matrix **X**_*g*|*g, h*_ represent the CV, where each column corresponds to the CV of each ROI's response to other ROIs within individual subjects' attention distributions, aggregated across all subjects. A high value for a certain ROI in **X**_*g*|*g, h*_ indicates that this ROI shows a unique pattern in the corresponding individual attention distribution matrix, contributing to classifying that individual into a specific group. Additionally, we compiled a list of the ROIs that rank in the top 25% based on both high averaged CV and high mean values in the attention distribution matrices ${\text{Y}}_{g|g,h}^{\left(i\right)}$ from group *g*. As shown in Figure 2H, the CV is calculated for each ROI within each subject, yielding a set of CV values for ROIs across all subjects. By averaging these values, we obtain the mean CV for each ROI. The top 25% ROIs with high mean values indicate frequent interactions with other ROIs, while those with high CV values suggest non-uniform signal patterns across subjects. Table 3 displays the top 25% unique ROIs for each group, compared between two disease groups-AD and MCI. Additional details on the top 25% ROIs for other group comparisons are provided in Supplementary Tables S2–S4.

**Table 3:** Top 25% ROIs that show differences between disease group of AD and MCI.

**Top**	**AD**	**MCI**
Top-1	Postcentral_L	Cingulum_Mid_L
Top-2	Postcentral_R	Postcentral_L
Top-3	Temporal_Inf_L	Fusiform_R
Top-4	Supp_Motor_Area_R	Precentral_R
Top-5	Fusiform_L	Pallidum_L
Top-6	Cingulum_Mid_L	Temporal_Inf_L
Top-7	Fusiform_R	Fusiform_L
Top-8	Cerebellum_8_R	Supp_Motor_Area_R
Top-9	Cerebellum_6_L	Insula_L
Top-10	Putamen_L	Cerebellum_6_R
Top-11	Cerebellum_8_L	Cerebellum_4_5_R
Top-12	Precentral_R	Postcentral_R
Top-13	Thalamus_L	Cerebellum_4_5_L
Top-14	Temporal_Mid_R	Cerebellum_6_L
Top-15	Cerebellum_4_5_R	Putamen_R
Top-16	Insula_R	Cerebellum_8_R
Top-17	Rolandic_Oper_R	Cerebellum_Crus2_R
Top-18	Precentral_L	SupraMarginal_R
Top-19	Temporal_Inf_R	Rolandic_Oper_R
Top-20	Insula_L	Hippocampus_R
Top-21	Putamen_R	Cingulum_Mid_R
Top-22	Cerebellum_7b_R	Pallidum_R
Top-23	Temporal_Mid_L	Thalamus_L
Top-24	Hippocampus_R	Putamen_L
Top-25	Cerebellum_4_5_L	Vermis_4_5
Top-26	Cerebellum_10_R	Paracentral_Lobule_L
Top-27	Cingulum_Mid_R	Vermis_8
Top-28	Pallidum_L	Precentral_L
Top-29	Cerebellum_7b_L	Supp_Motor_Area_L

Specific ROIs can be selected based on expert knowledge such as known disease mechanisms. This approach can facilitate deeper and more focused inferences when comparing ROIs between studied diseases. However, our fusion analytical framework provides a robust solution applicable to general resting-state fMRI datasets without requiring expert knowledge. Therefore, we selected ROIs based on statistical measures, such as the mean and the coefficient of variation in the attention distribution matrices. Without applying weights to the mean and coefficient of variation, we select the first quartile, representing the top 25% in both statistical measures. Using specific percentiles is a common approach in statistical analysis to highlight significant patterns and features within a dataset (Hastie, 2009; Silverman, 2018). Based on this approach, several previous studies have selected the top 25% features to identify distinctive patterns within data (Subramanian et al., 2005; Love et al., 2014). Similarly, we selected the top 25% of ROIs ranked by their mean and coefficient of variation.

### 3.3 Step 3: group representative features using the Latent Space Item-Response Model

In Step 2, we obtain the group representative matrices **X**_*h*|*g, h*_ for *g*≠*h*. To capture the group representative ROIs features that commonly reacted among subjects as shown in Figure 2I, we applied LSIRM to each group representative matrix ${\text{X}}_{h|g,h}\in {\mathbb{R}}^{N_{h}\times R}$. We estimated the latent positions of ROIs *V* = {**v**_*i*_}, for *i* = 1, ⋯ , 116 using Markov chain Monte Carlo (MCMC). The MCMC ran 55,000 iterations, and the first 5,000 iterations were discarded as burn-in processes. Then, from the remaining 50,000 iterations, we collected 10,000 samples with a thinning interval of 5. We used two-dimensional Euclidean space to estimate the latent positions of ROIs. Additionally, we set 0.005 for **β** jumping rule, 0.005 for **θ** jumping rule, and 0.005 for **w**_*j*_ and 0.003 **z**_*i*_ jumping rules. Here, we fixed the prior **β** to follow *N*(0, 1). We set *a*_σ_ = *b*_σ_ = 0.001. LSIRM takes each matrix **X**_*h*_ as input and provides the **O**_*h*_ matrix as output after the Procrustes-matching within the model. Since we calculated topics' distance on the 2-dimensional Euclidean space, **O**_*h*_ is of dimension 116 × 2. To overcome the identifiable issues from the invariance property, we applied oblimin rotation to the estimated topic position matrix ${\text{O}}_{h\%}^{*}$ using the R package GPArotation (https://cran.r-project.org/web/packages/GPArotation/index.html).

**Figure 6:**
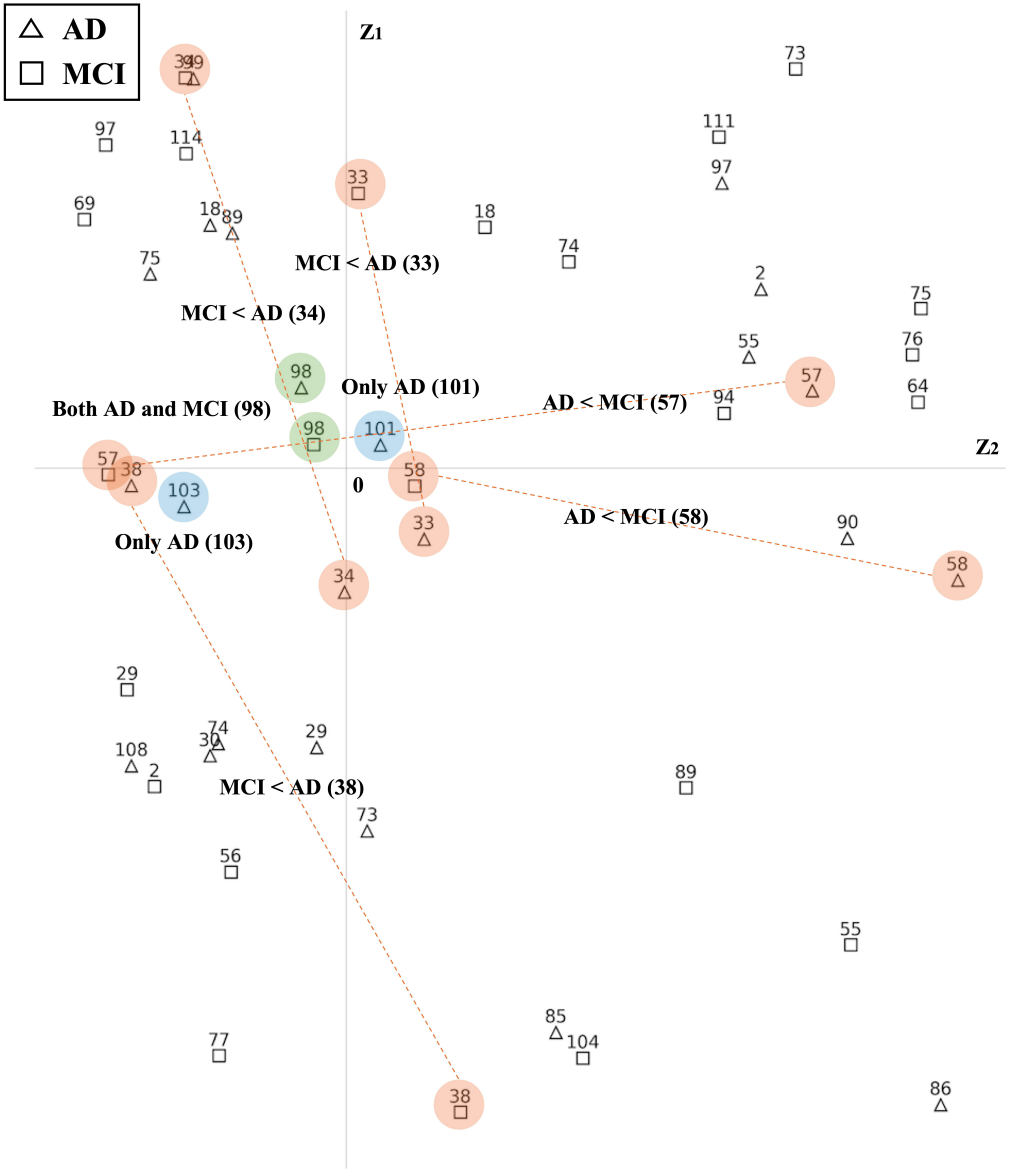
Latent positions of ROIs in 2-dimensional Euclidean space. The triangles indicate the top 25% ROIs from the AD group and the squares indicate the top 25% ROIs from the MCI group. Latent positions that are located closer to the origin suggest a higher likelihood of common interactions among subjects within the group. There are three scenarios: (1) a more reactive pattern, where, when comparing two groups, only the latent position of one group is located near the origin while the other group's latent position is situated outside the origin(orange color); (2) both group, where latent positions of both groups are near the origin (green color); and (3) only, indicating that a specific ROI is ranked in the top 25% within one group (blue color).

Based on their estimated latent positions, we successfully identified ROIs that exhibited common reactions to groups. Figure 6 and Supplementary Figures S7–S9 exclusively display the latent positions of the top 25% ROIs from each group. As depicted in Figure 6, the latent positions of ROIs are visualized in Euclidean space. By comparing the latent positions of ROIs from the two groups, we identified the ROIs exhibiting distinct patterns. In this representation, red-colored numbers signify the Top 25% ROIs from the AD group, while blue-colored numbers correspond to the top 25% ROIs from the MCI group. Notably, ROIs positioned closer to the origin in the latent space suggest a heightened likelihood of shared interactions among subjects within the same group. For instance, the latent position of 98 ROI from the AD and MCI groups is both located close to the origin and marked in green, indicating their significant roles in both AD and MCI. On the other hand, the latent positions of 101 and 103 ROIs are exclusively part of the AD group's top 25%. Moreover, other numbers highlighted in orange, indicate that only one ROI group possesses latent positions near the origin. These ROIs can be interpreted as significant features that exhibit meaningful reactions exclusively compared to the other group.

In our analysis framework, therefore, we specifically focus on the ROIs that meet two criteria: being ranked in the top 25% listed in Step 2 and having latent positions near the origin. These criteria indicate that these ROIs exhibit distinct patterns among subjects and can be considered representative features of each group. This selection process helps us identify the main features that are prominent and generalized well across the groups. We visually highlight these selected ROIs on the summary FCN from each group. As shown in Figure 2J, the summary FCN for each group is obtained by averaging the connectivity of each node across all subjects within the group. A threshold of 0.2, representing the top 5% connectivity ratios among the subjects' FCNs in each group, is then applied to define the connections. We have added histogram figures showing each group's connectivity ratio in Supplementary Figure S10.

**Figure 7:**
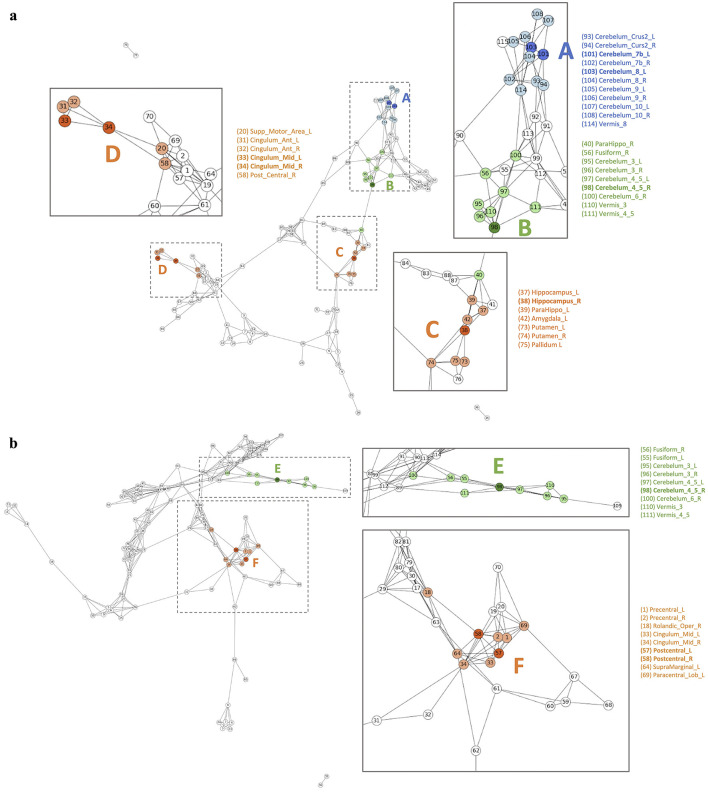
**(A)** AD group summary FCN and **(B)** MCI group summary FCN.

Figure 7 and Supplementary Figures S11–S13 show the differences in disease network between the two groups. The blue color indicates meaningful regions that show distinctive patterns from the attention distribution matrix compared to the other disease groups. The Orange color, on the other hand, indicates ROIs that were selected before analysis to be meaningful in both disease groups but were shown to only be meaningful in one group post-analysis. Finally, green indicates regions that were meaningful in both disease groups.

### 3.4 Interpretation of summary FCN from each group

Figure 7 and Supplementary Figure S11-S13 show the differences in disease network between the two groups. ROIs colored in blue indicate their selection as the top 25% group from the attention distribution matrix in one group, yet they do not appear as prominently significant in another group. Orange-colored ROIs indicate that they are meaningful only in one group, as revealed by the comparison between latent positions from each group. Finally, Green colored ROIs indicate that they were found to be meaningful in both disease groups. Utilizing the property of latent positions estimated from LSIRM (Jeon Y. et al., 2021), we managed to decode the structural connections among ROIs and identify ROIs that exhibited consistent significance across all subjects within each disease group. To see the overall connectivity, we merged the outcomes of LSIRM with FCNs, assigning colors to the significant ROIs and their interconnectedness with other ROIs in FCNs. The higher saturation colors indicate meaningful ROI features from LSIRM, and the same color with a lower brightness level reveals a direct connection from the meaningful ROI feature. We can regard these connections as a cluster.

#### 3.4.1 AD/MCI

Figure 7 illustrates the differences in the disease network between the AD group and MCI group, and Table 4 summarizes the meaningful ROIs. According to Figure 7A, Cluster A is comprised of Cerebellum_7_L (101) and Cerebellum_8_L (103). These two regions did not show activity in MCI, and the majority of regions that reacted in AD were connected to the Cerebellum regions. This distinction becomes evident as the AD group exhibits a diminished gray matter volume in the cerebellar anterior lobe in contrast to the non-AD group, as observed in prior research (Reiman et al., 2012).

**Table 4:** Meaningful ROIs in the comparison between the AD group and the MCI group.

**ROIs**	**AD**	**MCI**
Cingulum_Mid_L	▴	∇
Cingulum_Mid_R	▴	∇
Hippocampus_R	▴	∇
Postcentral_L	∇	▴
Postcentral_R	∇	▴
Cerebellum_4_5_R	▴	▴
Cerebellum_7_L	▴	−
Cerebellum_8_L	▴	−

▴ indicates regions that are active in the group, while ∇ represents regions that are less active than the other group. − indicates ROIs that are not selected as active in the group.

Cluster C (orange-colored cluster) with Hippocampus_R (38) and cluster D (orange-colored cluster) with Cingulum_Mid_L (33) and Cingulum_Mid_R (34) of Figure 7A show the cluster of regions and their direct connectivity that were more reactive in AD compared to MCI. Hippocampus_R (38) of cluster C showed greater reactivity in AD when compared to MCI and Hippocampus (37, 38), ParaHippo (39, 40), Putamen (73, 74), Pallidum (75, 76), and Amygdala (41, 42) are densely populated in this area. We discovered that the Hippocampus (37, 38) plays an important role in expressing AD characteristics (Figure 7A Cluster C). Many studies have shown that having Hippocampus (37, 38) dysfunction affects memory (Spaniol et al., 2009; Small et al., 2011; Delbeuck et al., 2003). Through our methodology, we also identified associations between the Hippocampus (37, 38), Putamen (73, 74), and ParaHippo (39, 40). These regions have been previously associated with cognitive impairment in Alzheimer's disease (Kesslak et al., 1991; Bobinski et al., 1999; de Jong et al., 2008).

Cluster F of Figure 7B shows ROIs, Postcentral_L (57) and Postcentral_R (58), that were more reactive in MCI compared to AD. This Postcentral (57, 58) is directly connected to Cingulum_Mid (33, 34), Precentral (1, 2), and Paracentral_Lob_L (69). According to our findings, Cingulum_Mid (33, 34) is linked to the Postcentral (57, 58), Precentral (1, 2), and Paracentral_Lob (69, 70), all of which are known to process motor information (Yeo et al., 2011).

Cluster B (green-colored cluster) of Figure 7A and Cluster E (green-colored cluster) of Figure 7B correspond to Cerebellum 4_5_R (98) that reacted to both AD and MCI. Both results show that Cerebellum 4_5_R (98) is not only connected with other Cerebellum regions but is also directly connected to Fusiform_L (55) and Fusiform_R (56), regions that are related to facial recognition (Kanwisher et al., 1997). According to the global hub node centrality analysis (Zhang et al., 2020), Fusiform (55, 56) and Cerebellum regions play important roles in constituting the key makeup of disease characteristics of MCI.

#### 3.4.2 AD/EMCI

Supplementary Figure S11 illustrates the differences in disease network between the AD group and the EMCI group, and Table 5 summarizes the meaningful ROIs. Clusters A and B of Figure S11A, which are Hippocampus_L (37), Lingual_R (48), Cerebellum_4_5_L (97), were found to be meaningful regions not in EMCI but only in AD. The hippocampus (37, 38) in both hemispheres is directly connected. These regions are also directly connected to ParaHippo (39, 40), Putamen (73, 74), Pallidum (75, 76), and Amygdala (41, 42) and the results are similar to the results described in Section 3.4.1. Fusiform_R (56)and Cerebellum_8_L (103) are directly connected to Hippocampus_R (38), and this is similar to cluster A and B of Supplementary Figure S11A. Hippocampus_L (37) and Lingual_R (48) are not only directly connected to Lingual_L (47), but also to Calcarine (43, 44), Cuneus (45, 46), Fusiform_L (56) and Cerebellum_6_L (99). Cluster D and E of Supplementary Figure S11A were more active in AD relative to EMCI and included the Hippocampus_R (38), Rolandic_Oper_R (18) regions. We can see that Rolandic_Oper_R (18) is directly connected to Putamen (73, 74), Pallidum (75, 76) and Heschl_L (79). Cluster F of Supplementary Figure S11B was active in EMCI but not AD, and Cerebellum_9_R (106) was analyzed. This region was adjacent to Cerebellum_Crus2_R (94), Cerebellum_7b_R (102), and Cerebellum_9_L (105). Cluster H, I, and J of Supplementary Figure S11B are regions that were more active in EMCI relative to AD, and regions Cingulum_Mid_L (33), Cingulum_Mid_R (34), Pallidum_L (75) and Cerebellum_Crus2_L (93) were analyzed. Cingulum_Mid is directly connected to Precentral (1, 2), Supp_Motor (19, 20), Postcentral (57, 58) and Supramarginal (63, 64). These connections have recently been examined in planning and cognitive control processing (Domic-Siede et al., 2021; Cavanagh and Frank, 2014).

**Table 5:** Meaningful ROIs in the comparison between the AD group and the EMCI group.

**ROIs**	**AD**	**EMCI**
Rolandic_Oper_R	▴	∇
Cingulum_Mid_L	∇	▴
Cingulum_Mid_R	∇	▴
Hippocampus_L	▴	−
Hippocampus_R	▴	∇
Lingual_R	▴	−
Fusiform_L	▴	▴
Fusiform_R	▴	▴
Pallidum_L	∇	▴
Cerebelm_Crus2_L	∇	▴
Cerebellum_4_5_L	▴	−
Cerebellum_9_R	−	▴

▴ indicates regions that are active in the group, while ∇ represents regions that are less active than the other group. − indicates ROIs that are not selected as active in the group.

Cluster C of Supplementary Figure S11A and Cluster G of Supplementary Figure S11B are regions that were active in both AD and EMCI, and correspond to Fusiform_L (55) and Fusiform_R (56). Fusiform (55, 56) is directly connected to Hippocampus (37, 38) and ParaHippo (39, 40). Likewise, in previous studies (Apostolova et al., 2006; Li et al., 2013; Zhu et al., 2009), there are connections between Hippocampus (37, 38) and ParaHippo (39, 40), Putamen (73, 74), Pallidum (75, 76), and Amgydala (41, 42).

#### 3.4.3 AD/LMCI

Supplementary Figure S12 illustrates the differences in disease network between the AD group and the LMCI group, and Table 6 summarizes the meaningful ROIs. Cluster A of Supplementary Figure S12A was active in AD but not LMCI, and Temporal_Mid_R(86) was analyzed. Cluster B of Supplementary Figure S12A reacted more in AD relative to LMCI, and Cerebellum_6_L (99) was analyzed. Not only is this region connected with multiple Cerebellum (91, 92, 100) areas, but it is also connected to Fusiform_L (55), Lingual (47, 48), and multiple Vermis (112, 113, 114). Cluster C of Supplementary Figure S12B was active only in LMCI and not AD, and Rolandic_Oper_R (18) was analyzed. This region was connected with Heschl (79, 80), Insula (29, 30) and Temporal_Sup (81, 82). Cluster D, E, and F of Supplementary Figure S12B are regions more active in LMCI relative to AD, and regions Putamen_L (73), Cerebellum_4_5_R (98) and Vermis_8 (114) were analyzed. Putamen (73, 74) is connected to Olfactory (21, 22), Hippocampus (37, 38), Amygdala (41, 42), Pallidum (75, 76) and Thalamus_L (77).

**Table 6:** Meaningful ROIs in the comparison between the AD group and the LMCI group.

**ROIs**	**AD**	**LMCI**
Rolandic_Oper_R	−	▴
Putamen_L	∇	▴
Temporal_Mid_R	▴	−
Cerebellum_4_5_R	∇	▴
Cerebellum_6_L	▴	∇
Vermis_8	∇	▴

▴ indicates regions that are active in the group, while ∇ represents regions that are less active than the other group. − indicates ROIs that are not selected as active in the group.

#### 3.4.4 EMCI/LMCI

Supplementary Figure S13 illustrates the differences in disease network between the EMCI group and the LMCI group, and Table 7 summarizes the meaningful ROIs. Clusters A, B, and C of Supplementary Figure S13A were regions that were only active in EMCI, and Frontal_Inf_Orb_R (16), Frontal_Med_Orb_R (26), and Cerebellum_3_R (96) were analyzed. Cerebellum_3_R (96) is directly connected to Cerebellum_3_L (95) and Vermis_3 (110). ROIs connected to Frontal_Inf_Orb (15, 16) and Frontal_Inf_Tri (13, 14), can be grouped as Frontal regions. We can also see that they are directly connected to Putamen_R (74). The Frontal_Med_Orb_R (26) is directly connected to Frontal_Med_Orb_L (25), Rectus (27, 28), and Frontal_Sup_Orb_R (6). Cluster D represents a region that was more active in EMCI relative to LMCI, including Putamen_L (73), and Pallidum_L (75) regions. The ROIs that were primarily connected to these regions can largely be defined as Caudate (71, 72), Pallidum_R (76), Thalamus (77, 78), Hippocampus (37, 38), and Insula (29, 30). Other regions include Rolandic_Oper_L (17), Amygdala_R (42), Fusiform_L (55), and Cerebellum_8_L (103). Supplementary Figure S13B, on the other hand, shows the FCN extracted for LMCI, which shows no significant ROIs that were significantly active only in LMCI. There is, however, cluster E, that shows ROIs more active in LMCI relative to EMCI. This cluster is comprised of ROIs connected to Temporal_Mid_R(86) and Cerebellum_6_L (99). Temporal_Mid_L (85) and Temporal_lnf_R(90) are ROIs connected with Temporal_Mid_R (86). ROIs connected with Cerebellum_6_L (99) are largely Fusiform (55, 56), Lingual (47, 48), and multiple Vermis (112, 113). Other regions include Cerebellum_Crus1_L (91), Cerebellum_4_5_L (97), and Cerebellum_6_R (100).

**Table 7:** Meaningful ROIs in the comparison between the EMCI group and the LMCI group.

**ROIs**	**EMCI**	**LMCI**
Frontal_Inf_Orb_R	▴	−
Frontal_Mid_Orb_R	▴	−
Putamen_L	▴	∇
Pallidum_L	▴	∇
Temporal_Mid_R	∇	▴
Cerebellum_3_R	▴	−
Cerebellum_6_L	∇	▴

▴indicates regions that are active in the group, while ∇ represents regions that are less active than the other group. − indicates ROIs that are not selected as active in the group.

## 4 Discussion

Our study presents a fusion analytic framework that provides interpretable distinctions in ROIs' connections between cognitive impairment groups. We construct FCNs to visualize intricate patterns within high-dimensional fMRI data and apply Self-Attn to capture hidden connectivity differences across groups. Our framework identifies group distinctive ROIs by summarizing individual-level attention distributions into group-level features. Using LSIRM, we model interactions among ROIs and estimate latent positions, providing intuitive insights into consistent group-specific responses. Finally, we highlight these distinctive ROIs within summary FCNs, offering deeper insights into the unique characteristics of each condition. Our framework can be extended to other neurodegenerative diseases by applying the same analytical approach to explore ROI connections and patterns, enabling the identification of distinctive neural characteristics across various conditions.

Furthermore, our fusion analytic framework can also be extended to multi-class classification. We can apply self-attention model to multi-class classification data. After obtaining each corresponding attention matrices, we estimate the latent positions of ROIs using LSIRM. As mentioned in Step 3, we can extract ROIs that display distinct patterns across different classes. In future work, we aim to expand this framework to multi-class classification, where distinct ROIs are not only differentiated between two groups but also across three or more disease groups.

Our fusion analytic framework has limitations in computing time. Since our framework involves three steps, fitting the data at each stage requires time, as each step depends on the previous one. Since the attention distribution output from Self-Attn influences the group representative matrix, which serves as the input for LSIRM where Self-Attn accuracy needs to be guaranteed. These sequential processes require users to input data and analyze results at each step, which can be more time-consuming than a single-stage model. Nonetheless, this multi-step approach is valuable as it enables users to check and interpret results at each stage.

Our methodology has also uncovered significant biological insights, which have been consistently validated across multiple studies. The four key features identified are (1) Hippocampus, (2) Cingulum, (3) Fusiform, and (4) Cerebellum.

When comparing AD with MCI, we found that the Hippocampus (37, 38) plays a crucial role in expressing AD characteristics (Figure 7A, Cluster C). The hippocampus is among the earliest regions to exhibit structural alterations in AD, with significant atrophy observed, particularly in subregions (Chételat et al., 2008). Numerous studies have demonstrated that dysfunction in the hippocampus (37, 38) negatively affects memory (Delbeuck et al., 2003; Spaniol et al., 2009; Small et al., 2011). Research has shown that disruption in connectivity between the hippocampus and parahippocampal cortex is correlated with memory dysfunction and cognitive decline (Sun et al., 2017).

Using our approach, we also identified connections between the Hippocampus (37, 38) and the Putamen (73, 74), as well as the Parahippocampal regions (39, 40), which have been associated with cognitive impairment in Alzheimer's disease (Kesslak et al., 1991; Bobinski et al., 1999; de Jong et al., 2008). The parahippocampal cortex has shown altered connectivity with the hippocampus in MCI and AD patients, which is linked to reduced cognitive performance. This region is essential for maintaining declarative memory, and disruptions here are associated with early cognitive decline in AD (Liu et al., 2016). The putamen, along with the thalamus, experiences significant atrophy in AD patients, with this volume reduction correlating with cognitive decline. This suggests that deep gray matter structures play a role in AD's neurodegenerative processes, extending beyond the traditional focus on the hippocampus (de Jong et al., 2008).

When compared to EMCI, the biomarkers of AD become more clearly evident (Supplementary Figure S11A). These biomarkers are detected in the connections between the Hippocampus (37, 38) and the ParaHippocampal regions (39, 40), Putamen (73, 74), Pallidum (75, 76), and Amygdala (41, 42) (Apostolova et al., 2006; Zhu et al., 2009; Li et al., 2013). Studies indicate that specific changes in these ROIs are significant as the condition progresses from EMCI to AD. For instance, multimodal imaging techniques have shown atrophy in the hippocampus and amygdala during the early stages of AD, which correlates strongly with cognitive decline (Eustache et al., 2016). Additionally, functional alterations in the connectivity between the hippocampus and parahippocampal regions have been associated with disrupted memory processing and the hallmark symptoms of AD (Wei et al., 2020). Moreover, the putamen and pallidum show progressive volume loss in patients transitioning from MCI to AD, which emphasizes their role in neurodegeneration (Yi et al., 2016). Collectively, these findings underline the interconnected nature of these regions in reflecting the underlying pathology of Alzheimer's disease as it progresses from EMCI.

In terms of the Cingulum, our method provides additional insights regarding the Cingulum_Mid (33, 34) to MCI, which has traditionally been considered one of the most important ROIs in AD (Yoshiura et al., 2002; Fellgiebel et al., 2005; Kantarci et al., 2000; Scheff and Price, 2001; Catheline et al., 2010). The Cingulum_Mid (33, 34) is responsible for processing motor and attention-related activities (Lin et al., 2014). However, our findings reveal that the Cingulum_Mid (33, 34) is more prominent in MCI and EMCI than in AD (Figure 7A, Supplementary Figure S11B). The Cingulum_Mid (33, 34) is connected to the Postcentral (57, 58), Precentral (1, 2), and Paracentral_Lob (69, 70) regions, all of which are involved in motor processing. These four regions have been investigated using fluorodeoxyglucose (FDG) positron emission tomography (PET) as relevant indicators in MCI (Xu et al., 2016). Furthermore, distinct patterns of atrophy and reduced fractional anisotropy values in these ROIs have been noted in neuroimaging studies, emphasizing their importance in predicting the progression from MCI to AD (Choo et al., 2010).

In the network comparison between EMCI and AD, our model identified a connection between the Cingulum_Mid (33, 34) and the Supramarginal gyrus (63, 64), which is associated with motor attention-related activity (Supplementary Figure S11B). These connections have recently been examined in relation to planning and cognitive control processes (Cavanagh and Frank, 2014; Domic-Siede et al., 2021). The Cingulum_Mid and Supramarginal gyrus have been implicated in age-related motor performance changes, highlighting their role in cognitive and motor integration (Heuninckx et al., 2005). As a result, the Cingulum_Mid (33, 34), which has previously been underexplored, should be considered relevant to both cognitive and motor functions in MCI and EMCI. These findings support its significance in understanding the progression of cognitive decline, especially as the interaction between these regions reflects the brain's adaptive changes in response to increasing cognitive demand.

On the other hand, in both AD and MCI, the Fusiform (55, 56) emerges as a key ROI in revealing the characteristics of each disease. The Fusiform (55, 56) is well known for its role in facial recognition processing (Kanwisher et al., 1997) and has recently been studied for its genetic and epigenetic links to AD (Ma et al., 2020). Our method identified the Fusiform (55, 56) as a significant ROI in both AD and EMCI (Supplementary Figure S11), showing that it is connected to cerebellar functions. This connection is consistently observed across all network results (Figure 7, Supplementary Figure S11–S13). Research also suggests that the cerebellum and fusiform gyrus exhibit strong interconnections, which play crucial roles in cognitive functions as MCI progresses toward AD (Yao et al., 2010).

Our research highlights the increasing significance of the cerebellum in the context of cognitive impairment. Traditionally, the cerebellum was associated primarily with motor control and coordination (Schmahmann et al., 2001; Aggarwal et al., 2006). However, recent studies have shown its involvement in cognitive processing and emotional modulation (Schmahmann, 1998; Schmahmann and Sherman, 1998; Whitwell et al., 2012) and emotional modulation (Schmahmann et al., 2007). Recently, the cerebellum has also been recognized as a useful biomarker in clinical AD subjects (Russo et al., 2018). According to our results, Cerebellum_4_5_R (98) plays a significant role as an ROI in both AD and MCI (Figure 7). This aligns with prior studies showing decreased activity in this region in both AD and MCI compared to the control group (Wang et al., 2011). Additionally, structural MRI studies have explored changes in the cerebellum, finding that the posterior cerebellar lobes were significantly smaller in AD patients and correlated with poorer cognitive performance, suggesting that cerebellar atrophy may contribute to AD progression(Thomann et al., 2008).

## Data Availability

The datasets analyzed during the current study are available in the Alzheimer's Disease Neuroimaging Initiative (ADNI) repository, https://adni.loni.usc.edu/. We have uploaded the core part of our method, to the following URL on Gitub: https://github.com/jeon9677/summary-fcn.
