# Supplementary material for: A fusion analytic framework for investigating functional brain connectivity differences using resting-state fMRI
Source: Front Neurosci. 2024 Dec 11;18:1402657. doi: 10.3389/fnins.2024.1402657 (PMC11668745; doi:10.3389/fnins.2024.1402657)
Supplement: Supplementary file 1 [file Data_Sheet_1.pdf]

Supplementary Material

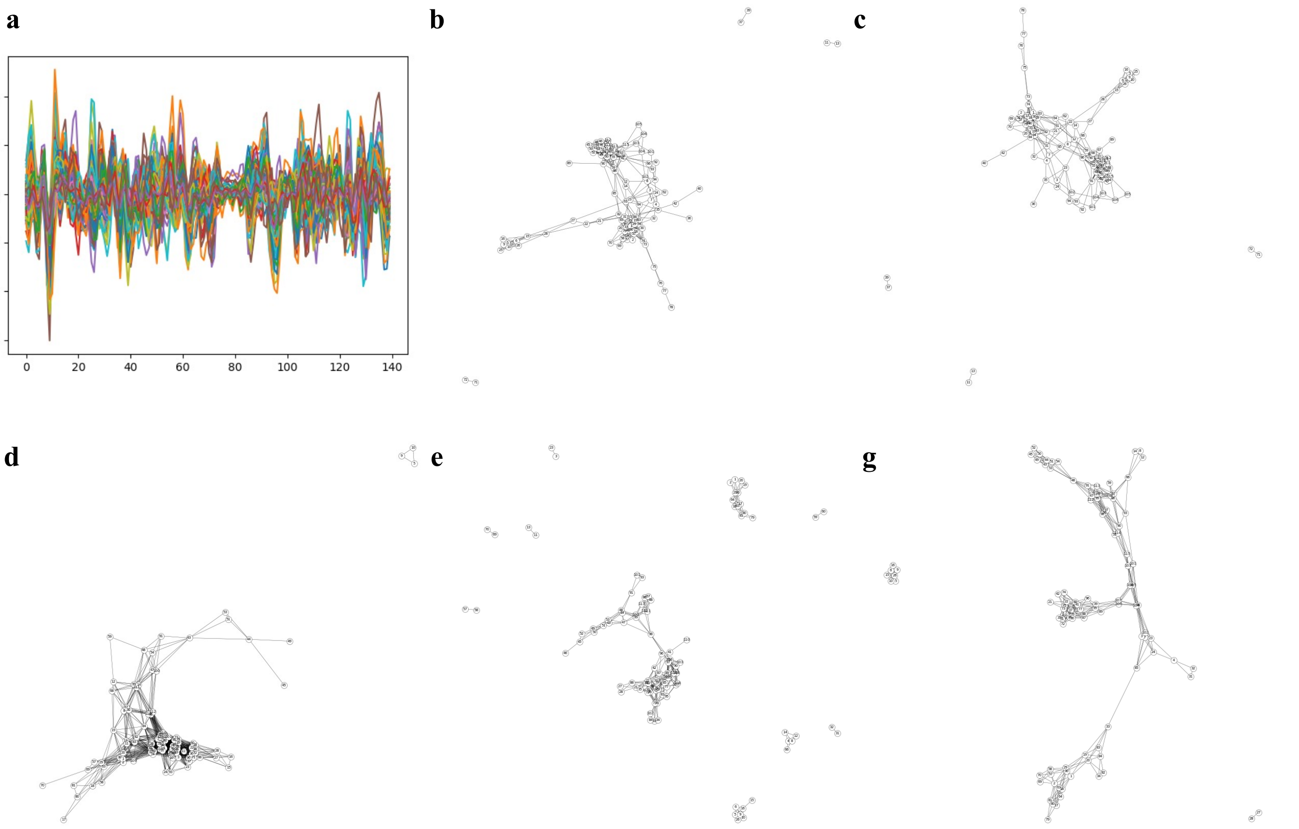

Figure S1. Correlation– and dimension reduction–based FCNs for an MCI subject. (**a**) shows ROIs and rs–fMRI BOLD signals for an MCI subject. FCNs were generated using correlation–based methods: Pearson's r and Fisher's z (shown in **b**, **c**). To better understand the interrelationships between brain regions, dimension reduction techniques were applied to estimate latent positions of ROIs: PCA in **d** (linear space), t–SNE in **e** (stochastic space), and UMAP in **f** (topological space).

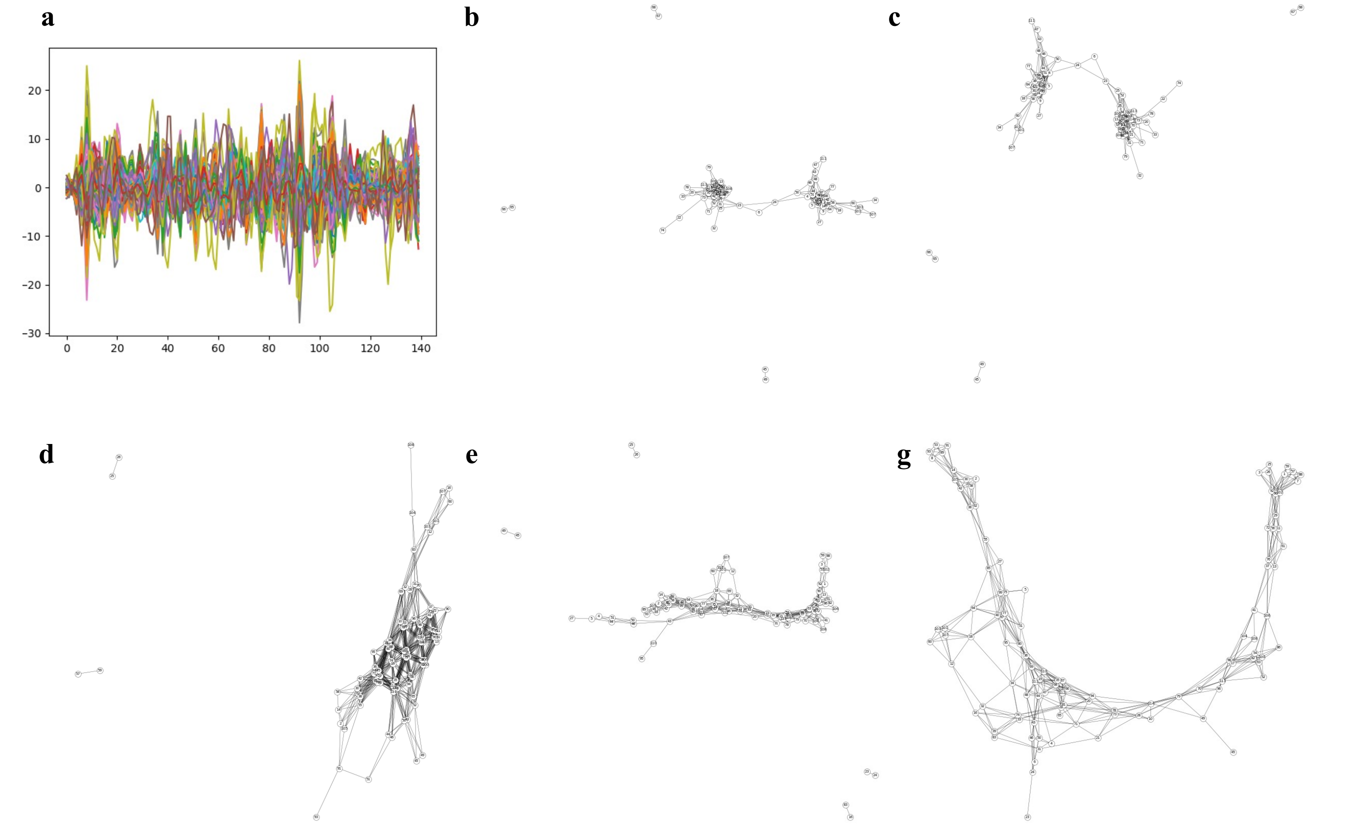

Figure S2. Correlation– and dimension reduction–based FCNs for an EMCI subject. (**a**) shows ROIs and rs–fMRI BOLD signals for an EMCI subject. FCNs were generated using correlation–based methods: Pearson's r and Fisher's z (shown in **b**, **c**). To better understand the interrelationships between brain regions, dimension reduction techniques were applied to estimate latent positions of ROIs: PCA in **d** (linear space), t–SNE in **e** (stochastic space), and UMAP in **f** (topological space).

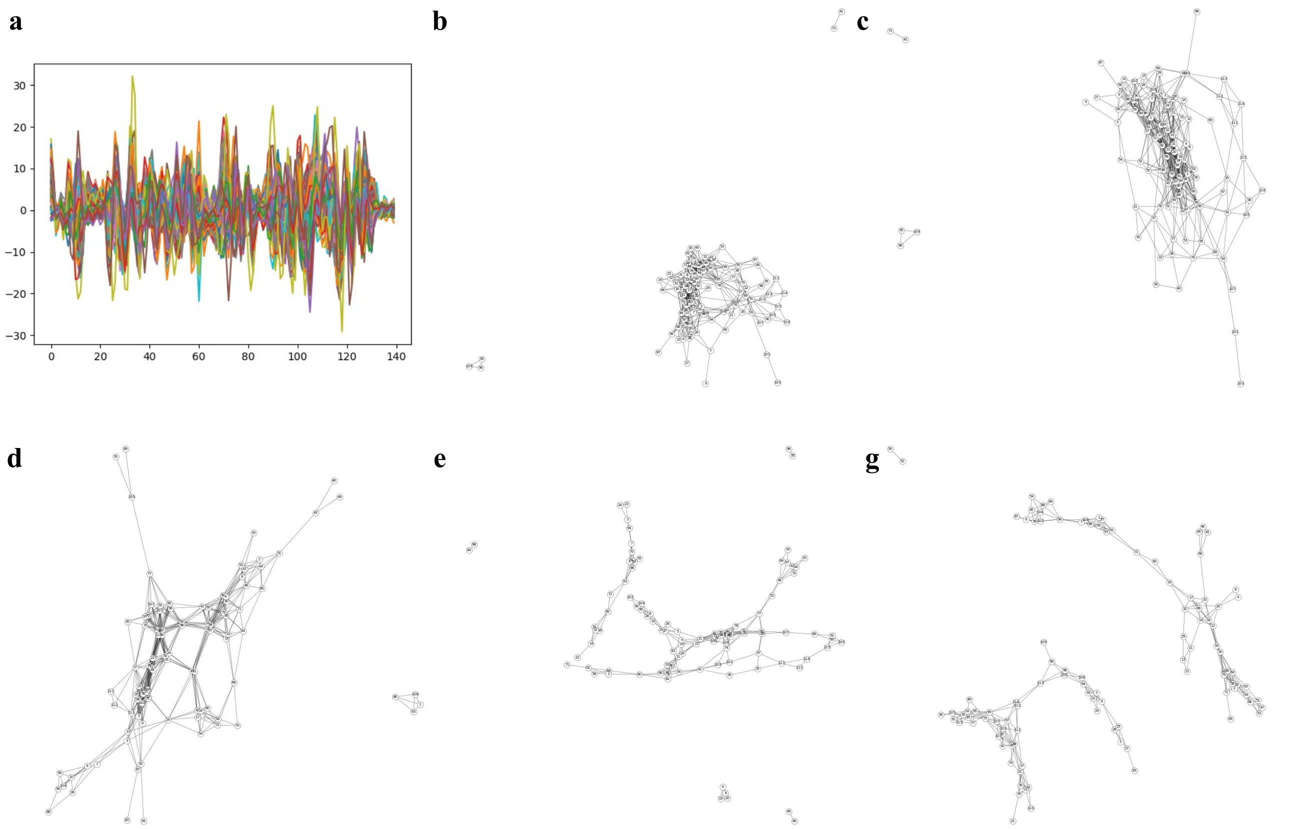

Figure S3. Correlation– and dimension reduction–based FCNs for an LMCI subject. (**a**) shows ROIs and rs–fMRI BOLD signals for an LMCI subject. FCNs were generated using correlation–based methods: Pearson's r and Fisher's z (shown in **b**, **c**). To better understand the interrelationships between brain regions, dimension reduction techniques were applied to estimate latent positions of ROIs: PCA in **d** (linear space), t–SNE in **e** (stochastic space), and UMAP in **f** (topological space).

#	Name	#	Name	#	Name	#	Name
1	Precentral_L	30	Insula_R	59	Parietal_Sup_L	88	Temp_Pole_Mid_R
2	Precentral_R	31	Cingulum_Ant_L	60	Parietal_Sup_R	89	Temporal_Inf_L
3	Frontal_Sup_L	32	Cingulum_Ant_R	61	Parietal_Inf_L	90	Temporal_Inf_R
4	Frontal_Sup_R	33	Cingulum_Mid_L	62	Parietal_Inf_R	91	Cerebelm_Crus1_L
5	Frontal_Sup_Orb_L	34	Cingulum_Mid_R	63	SupraMarginal_L	92	Cerebelm_Crus1_R
6	Frontal_Sup_Orb_R	35	Cingulum_Post_L	64	SupraMarginal_R	93	Cerebelm_Crus2_L
7	Frontal_Mid_L	36	Cingulum_Post_R	65	Angular_L	94	Cerebelm_Crus2_R
8	Frontal_Mid_R	37	Hippocampus_L	66	Angular_R	95	Cerebelum_3_L
9	Frontal_Mid_Orb_L	38	Hippocampus_R	67	Precuneus_L	96	Cerebelum_3_R
10	Frontal_Mid_Orb_R	39	ParaHippo_L	68	Precuneus_R	97	Cerebelum_4_5_L
11	Frontal_Inf_Oper_L	40	ParaHippo_R	69	Paracentral_Lob_L	98	Cerebelum_4_5_R
12	Frontal_Inf_Oper_R	41	Amygdala_L	70	Paracentral_Lob_R	99	Cerebelum_6_L
13	Frontal_Inf_Tri_L	42	Amygdala_R	71	Caudate_L	100	Cerebelum_6_R
14	Frontal_Inf_Tri_R	43	Calcarine_L	72	Caudate_R	101	Cerebelum_7_L
15	Frontal_Inf_Orb_L	44	Calcarine_R	73	Putamen_L	102	Cerebelum_7_R
16	Frontal_Inf_Orb_R	45	Cuneus_L	74	Putamen_R	103	Cerebelum_8_L
17	Rolandic_Oper_L	46	Cuneus_R	75	Pallidum_L	104	Cerebelum_8_R
18	Rolandic_Oper_R	47	Lingual_L	76	Pallidum_R	105	Cerebelum_9_L
19	Supp_Motor_L	48	Lingual_R	77	Thalamus_L	106	Cerebelum_9_R
20	Supp_Motor_R	49	Occipital_Sup_L	78	Thalamus_R	107	Cerebelum_10_L
21	Olfactory_L	50	Occipical_Sup_R	79	Heschl_L	108	Cerebelum_10_R
22	Olfactory_R	51	Occipital_Mid_L	80	Heschl_R	109	Vermis_1_2
23	Frontal_Sup_Med_L	52	Occipical_Mid_R	81	Temporal_Sup_L	110	Vermis_3
24	Frontal_Sup_Med_R	53	Occipital_Inf_L	82	Temporal_Sup_R	111	Vermis_4_5
25	Frontal_Mid_Orb_L	54	Occipital_Inf_R	83	Templ_Pole_Sup_L	112	Vermis_6
26	Frontal_Mid_Orb_R	55	Fusiform_L	84	Templ_Pole_Sup_R	113	Vermis_7
27	Rectus_L	56	Fusiform_R	85	Temporal_Mid_L	114	Vermis_8
28	Rectus_R	57	Postcentral_L	86	Temporal_Mid_R	115	Vermis_9
29	Insula_L	58	Postcentral_R	87	Templ_Pole_Mid_L	116	Vermis_10

Table S1. Automated anatomical labeling–116 template

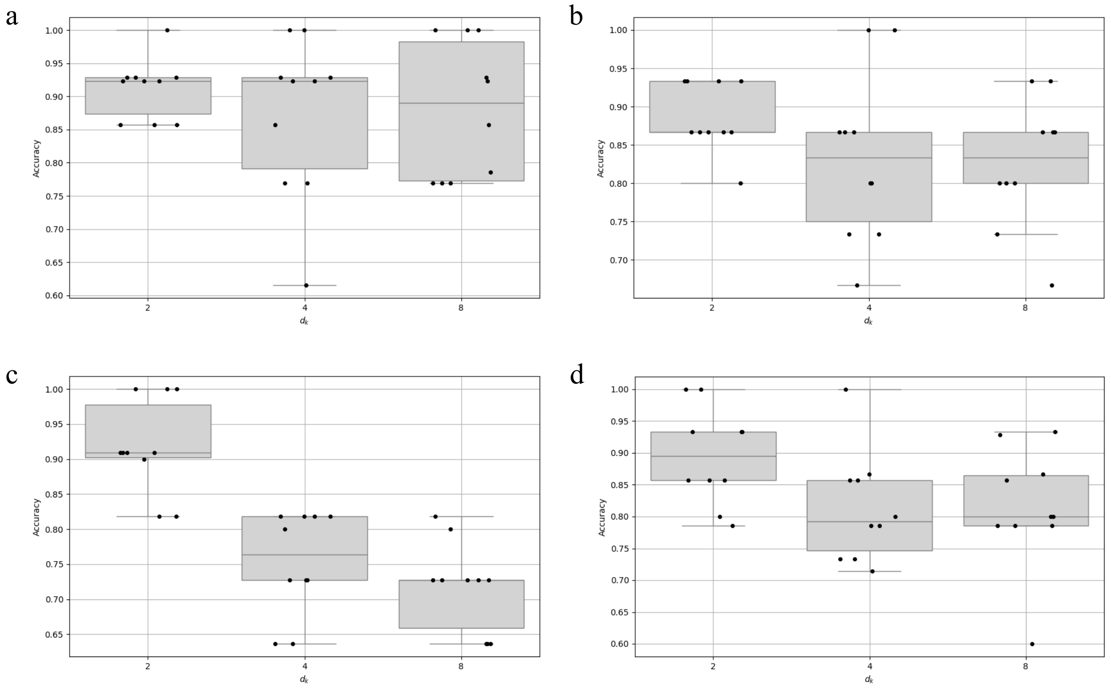

Figure S4. The classification accuracy results using 10-fold cross–validation for different values of d_k (2, 4, and 8 where $h = 128$) in the proposed Self–Attn model. Figures (a) through (d) show the accuracy for the following classification tasks: (a) AD vs. MCI, (b) AD vs. EMCI, (c) AD vs. LMCI, and (d) EMCI vs. LMCI. Each dot represents the accuracy of an individual fold. The interquartile ranges and medians are shown for each d_k value. The configuration with $d_k = 2$ consistently demonstrated higher accuracy and lower variability, indicating greater robustness compared to $d_k = 4$ and $d_k = 8$. Based on these findings, $d_k = 2$ was selected for optimal model performance.

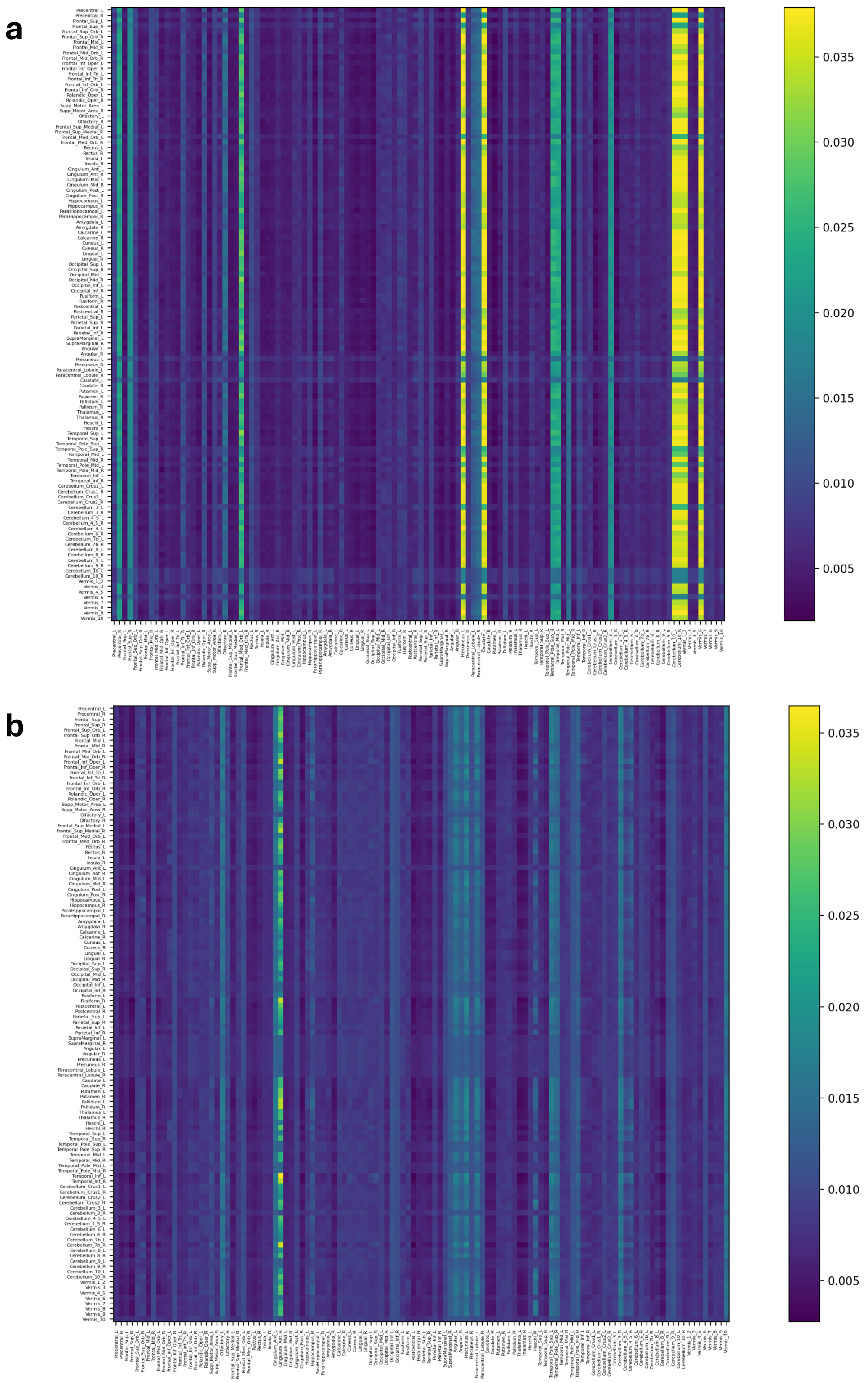

Figure S5. Attention distribution matrix of two subjects within the AD group, generated by Self–Attn designed for AD and MCI classification, utilizing topological–based FCNs as input.

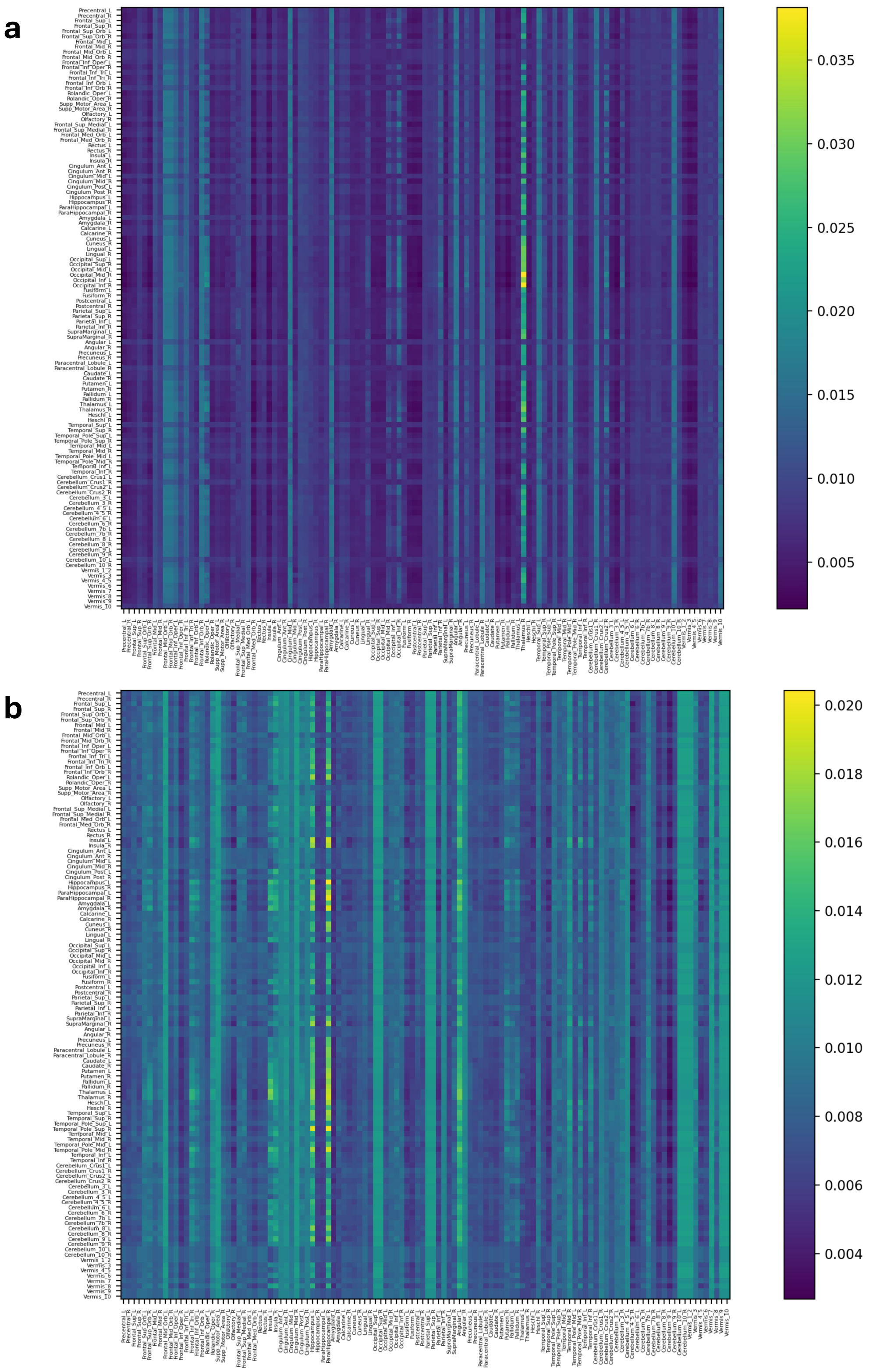

Figure S6. Attention distribution matrix of two subjects within the MCI group, generated by Self–Attn designed for AD and MCI classification, utilizing topological–based FCNs as input.

Top	AD	EMCI
Top–1	Fusiform_R	Fusiform_L
Top–2	Cerebelum_6_L	Temporal_Inf_L
Top–3	Pallidum_R	Cingulum_Mid_L
Top–4	Postcentral_L	Pallidum_R
Top–5	Fusiform_L	Fusiform_R
Top–6	Cerebelum_6_R	Frontal_Inf_Orb_L
Top–7	Hippocampus_R	Cerebelum_6_L
Top–8	Cingulum_Mid_L	Frontal_Inf_Tri_R
Top–9	Cerebelum_7b_L	Putamen_R
Top–10	Putamen_R	Cingulum_Mid_R
Top–11	Cerebelum_4_5_L	Cerebelum_6_R
Top–12	Temporal_Inf_L	Temporal_Inf_R
Top–13	Cingulum_Mid_R	Vermis_8
Top–14	Cerebelum_8_L	Insula_L
Top–15	Hippocampus_L	Pallidum_L
Top–16	Rolandic_Oper_R	Frontal_Sup_R
Top–17	Pallidum_L	Cerebelum_9_R
Top–18	Cerebelum_Crus2_L	Temporal_Sup_R
Top–19	Cerebelum_7b_R	Postcentral_L
Top–20	Putamen_L	Cerebelum_7b_L
Top–21	Temporal_Inf_R	Cerebelum_8_R
Top–22	Insula_L	Rolandic_Oper_L
Top–23	Cerebelum_Crus2_R	Putamen_L
Top–24	Rolandic_Oper_L	Rolandic_Oper_R
Top–25	Cerebelum_8_R	Insula_R
Top–26	Cerebelum_3_L	Cerebelum_Crus2_L
Top–27	Frontal_Inf_Tri_R	Cerebelum_3_L
Top–28	Frontal_Sup_R	Frontal_Inf_Tri_L
Top–29	Lingual_R	Hippocampus_R

Table S2. Top 25% ROIs that show differences between disease group of AD and EMCI.

Top	AD	LMCI
Top–1	Pallidum_L	Cerebelum_8_L
Top–2	Putamen_L	Cerebelum_8_R
Top–3	Cerebelum_8_L	Pallidum_R
Top–4	Cerebelum_8_R	Putamen_L
Top–5	Putamen_R	Pallidum_L
Top–6	Pallidum_R	Putamen_R
Top–7	Cerebelum_6_L	Hippocampus_R
Top–8	Cerebelum_7b_R	Vermis_8
Top–9	Fusiform_L	Cerebelum_7b_L
Top–10	Insula_R	Cerebelum_6_L
Top–11	Cerebelum_Crus2_R	Cerebelum_4_5_R
Top–12	Vermis_8	Fusiform_R
Top–13	Vermis_7	Cingulum_Mid_R
Top–14	Cerebelum_9_R	Vermis_6
Top–15	Lingual_R	Cerebelum_9_R
Top–16	Cerebelum_6_R	Thalamus_L
Top–17	Hippocampus_R	Cerebelum_7b_R
Top–18	Fusiform_R	Temporal_Mid_R
Top–19	Cerebelum_4_5_R	Cerebelum_6_R
Top–20	Cerebelum_9_L	Cerebelum_4_5_L
Top–21	Cerebelum_7b_L	Vermis_7
Top–22	Insula_L	Cerebelum_Crus2_R
Top–23	Olfactory_L	Insula_L
Top–24	Rolandic_Oper_L	Olfactory_R
Top–25	Olfactory_R	Rectus_L
Top–26	Precuneus_L	Temporal_Inf_R
Top–27	Cerebelum_Crus1_L	Temporal_Mid_L
Top–28	Rolandic_Oper_R	Rolandic_Oper_L
Top–29	Thalamus_L	Lingual_R

Table S3. Top 25% ROIs that show differences between disease group of AD and LMCI.

Top	EMCI	LMCI
Top–1	Putamen_L	Putamen_L
Top–2	Putamen_R	Fusiform_R
Top–3	Pallidum_R	Pallidum_L
Top–4	Pallidum_L	Putamen_R
Top–5	Temporal_Inf_L	Hippocampus_R
Top–6	Fusiform_L	Pallidum_R
Top–7	Fusiform_R	Fusiform_L
Top–8	Temporal_Inf_R	Hippocampus_L
Top–9	Hippocampus_R	Temporal_Inf_R
Top–10	Insula_L	Temporal_Inf_L
Top–11	Olfactory_R	Cingulum_Mid_R
Top–12	Rolandic_Oper_R	Thalamus_L
Top–13	Cerebelum_6_L	Cerebelum_6_L
Top–14	Temporal_Mid_R	Insula_R
Top–15	Insula_R	Cerebelum_4_5_L
Top–16	Frontal_Inf_Orb_R	Cerebelum_8_L
Top–17	Hippocampus_L	Olfactory_R
Top–18	Rolandic_Oper_L	Insula_L
Top–19	Cerebelum_7b_R	Cingulum_Mid_L
Top–20	Cerebelum_7b_L	Cerebelum_6_R
Top–21	Cingulum_Mid_R	Rolandic_Oper_R
Top–22	ParaHippo_R	Precentral_L
Top–23	Cerebelum_3_R	ParaHippo_R
Top–24	Thalamus_R	Cerebelum_4_5_R
Top–25	Thalamus_L	Cerebelum_7b_R
Top–26	Olfactory_L	Thalamus_R
Top–27	Cingulum_Mid_L	ParaHippo_L
Top–28	Frontal_Med_Orb_R	Temporal_Mid_R
Top–29	Cerebelum_4_5_R	Amygdala_L

Table S4. Top 25% ROIs that show differences between disease group of EMCI and LMCI.

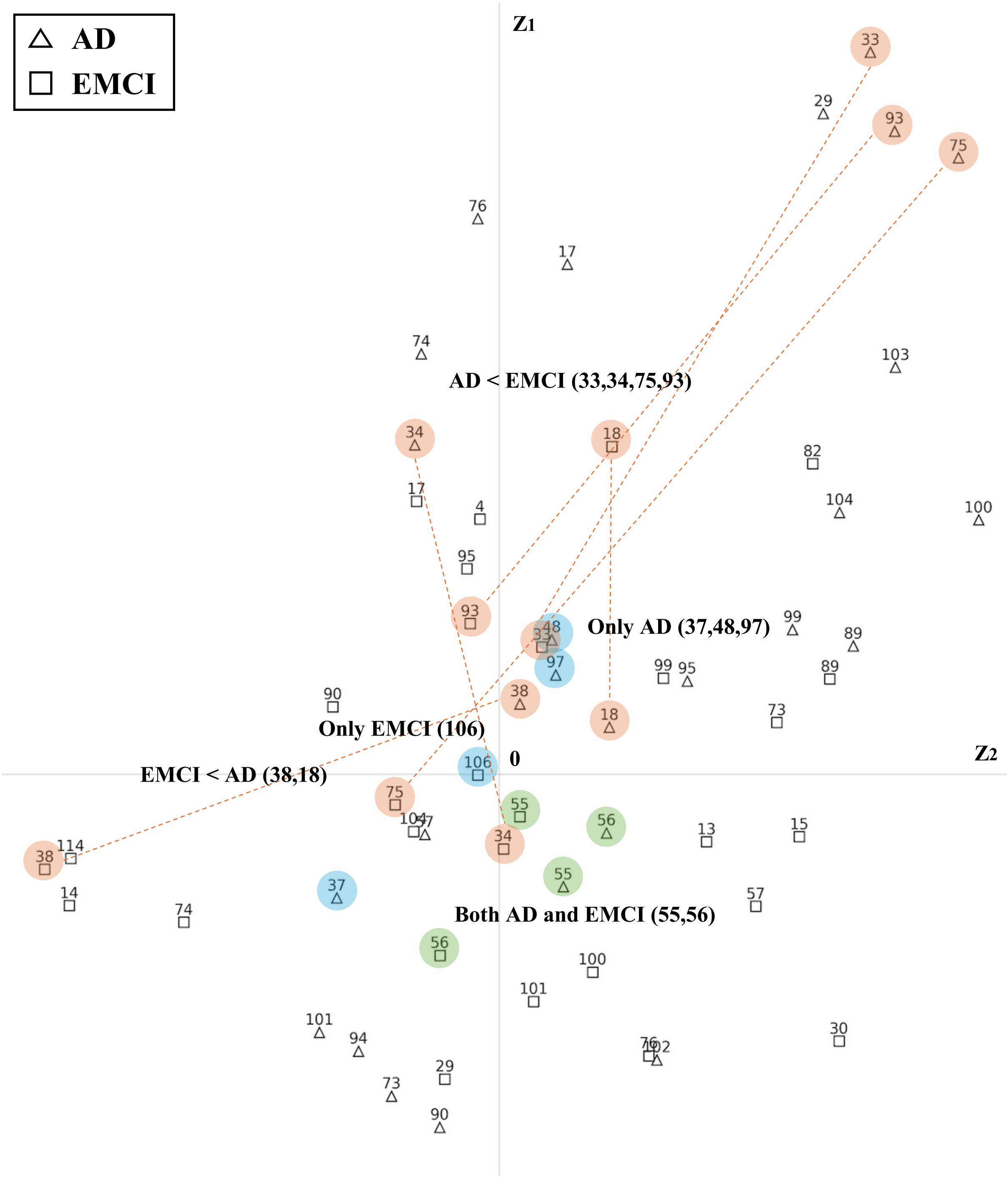

Figure S7. Latent positions of ROIs in 2–dimensional Euclidean space. The triangles indicate the top 25% ROIs from AD group and the squares indicate top 25% ROIs from EMCI group. Latent positions located closer to the origin suggest a higher likelihood of common interactions among subjects within the group. There are three scenarios: (1) a more reactive pattern, where, when comparing two groups, only the latent position of one group is located near the origin while the other group's latent position is situated outside the origin(orange color); (2) both group, where latent positions of both groups are near the origin (green color); and (3) only, indicating that a specific ROI is ranked in the top 25% within one group (blue color).

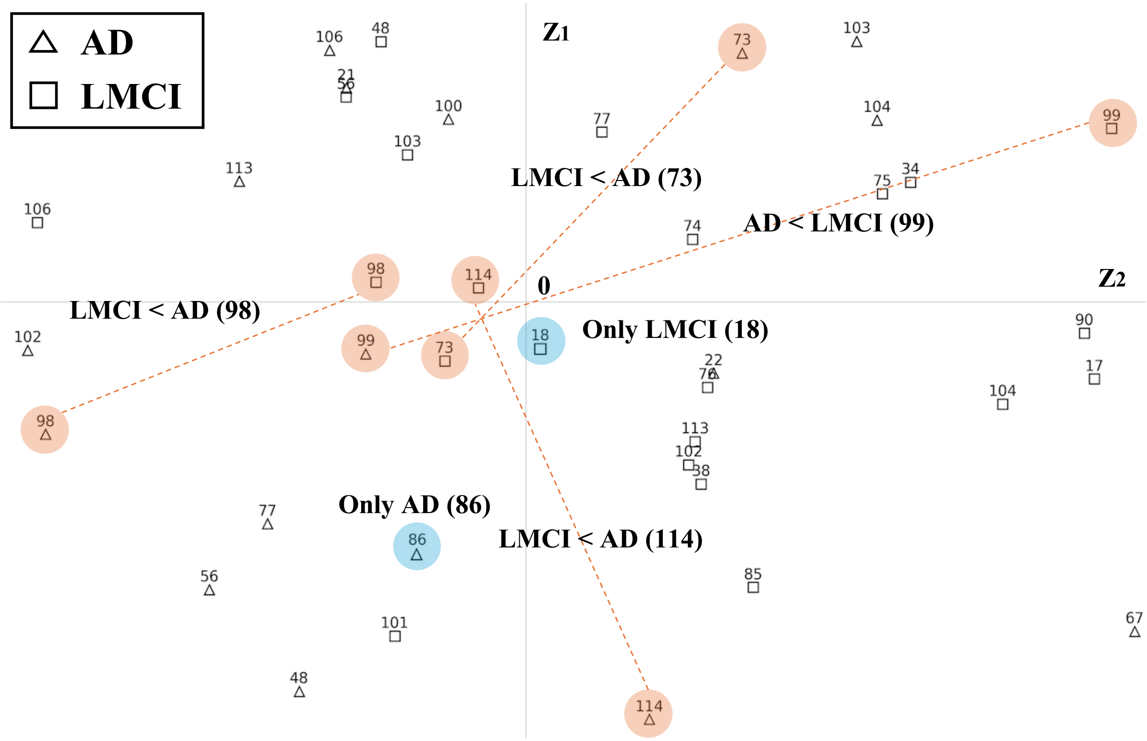

Figure S8. Latent positions of ROIs in 2–dimensional Euclidean space. The triangles indicate Top 25% ROIs from AD group and the squares indicate top 25% ROIs from LMCI group. Latent positions located closer to the origin suggest a higher likelihood of common interactions among subjects within the group. There are three scenarios: (1) more reactive pattern, where when comparing two groups, only the latent position of one group is located near the origin while the other group's latent position is situated outside the origin(orange color); (2) both group, where latent positions of both groups are near the origin (green color); and (3) only, indicating that a specific ROI is ranked in the top 25% within one group (blue color).

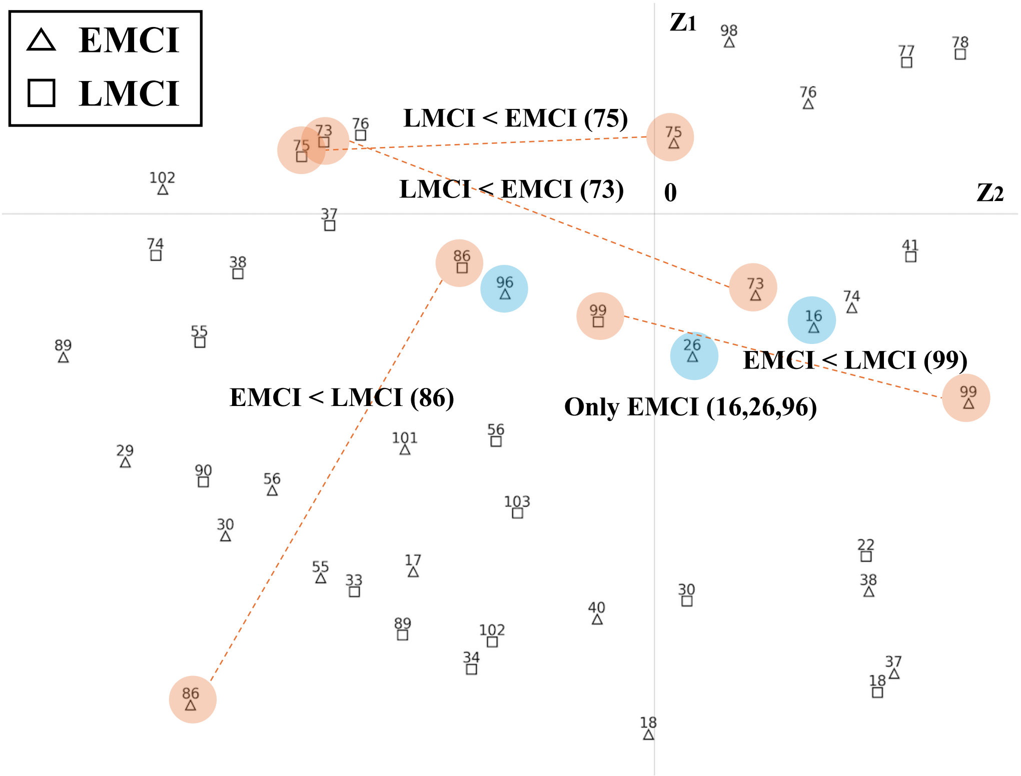

Figure S9. Latent positions of ROIs in 2–dimensional Euclidean space. The triangles indicate the top 25% ROIs from EMCI group and the squares indicate top 25% ROIs from LMCI group. Latent positions located closer to the origin suggest a higher likelihood of common interactions among subjects within the group. There are three scenarios: (1) a more reactive pattern, where, when comparing two groups, only the latent position of one group is located near the origin while the other group's latent position is situated outside the origin(orange color); (2) both group, where latent positions of both groups are near the origin (green color); and (3) only, indicating that a specific ROI is ranked in the top 25% within one group (blue color).

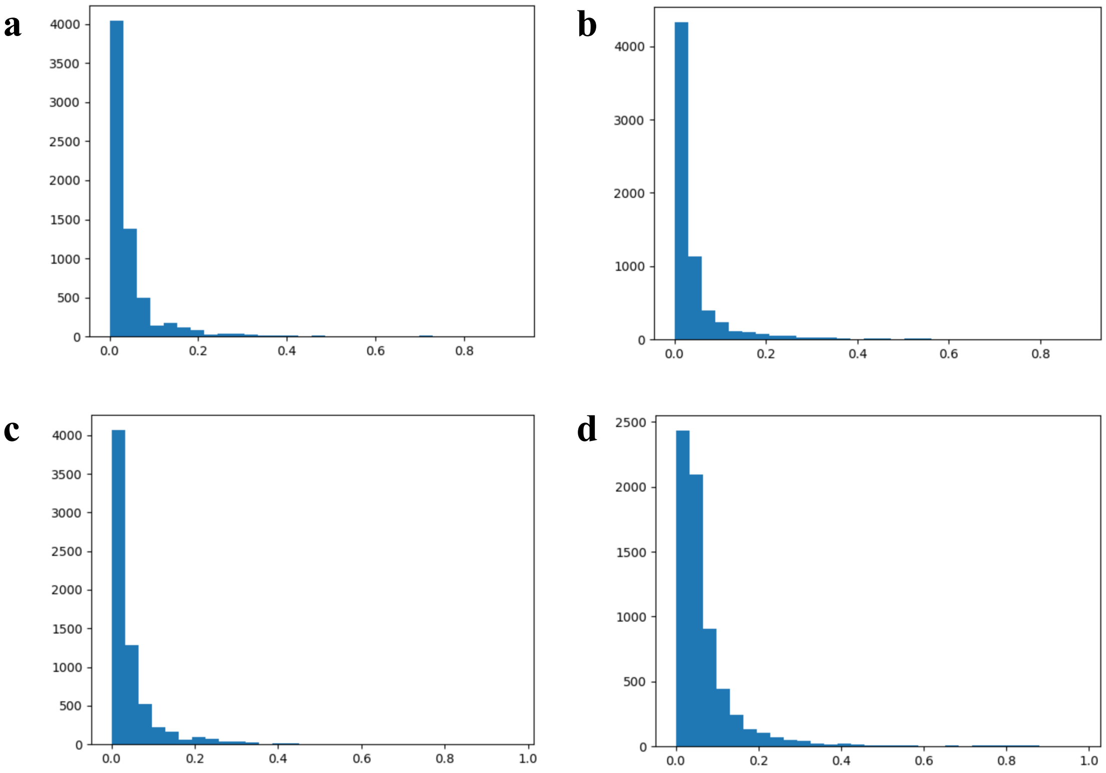

Figure S10. Connection ratios distribution between ROIs within FCNs for each group: (a) AD group based on UMAP, (b) MCI group based on UMAP, (c) AD group based on t-SNE, and (d) EMCI group based on t-SNE.

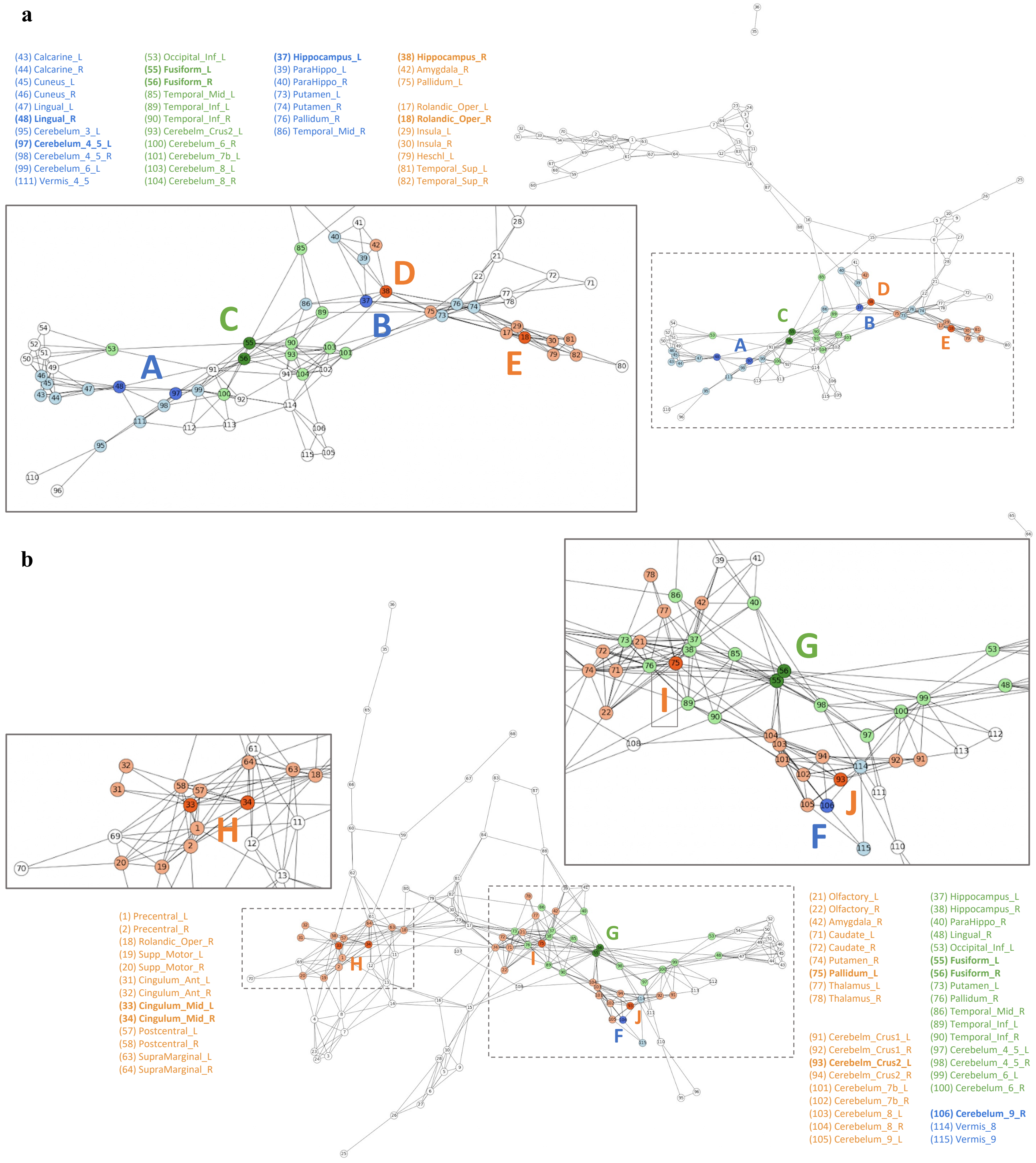

Figure S11. **(a)** AD group summary FCN and **(b)** EMCI group summary FCN.

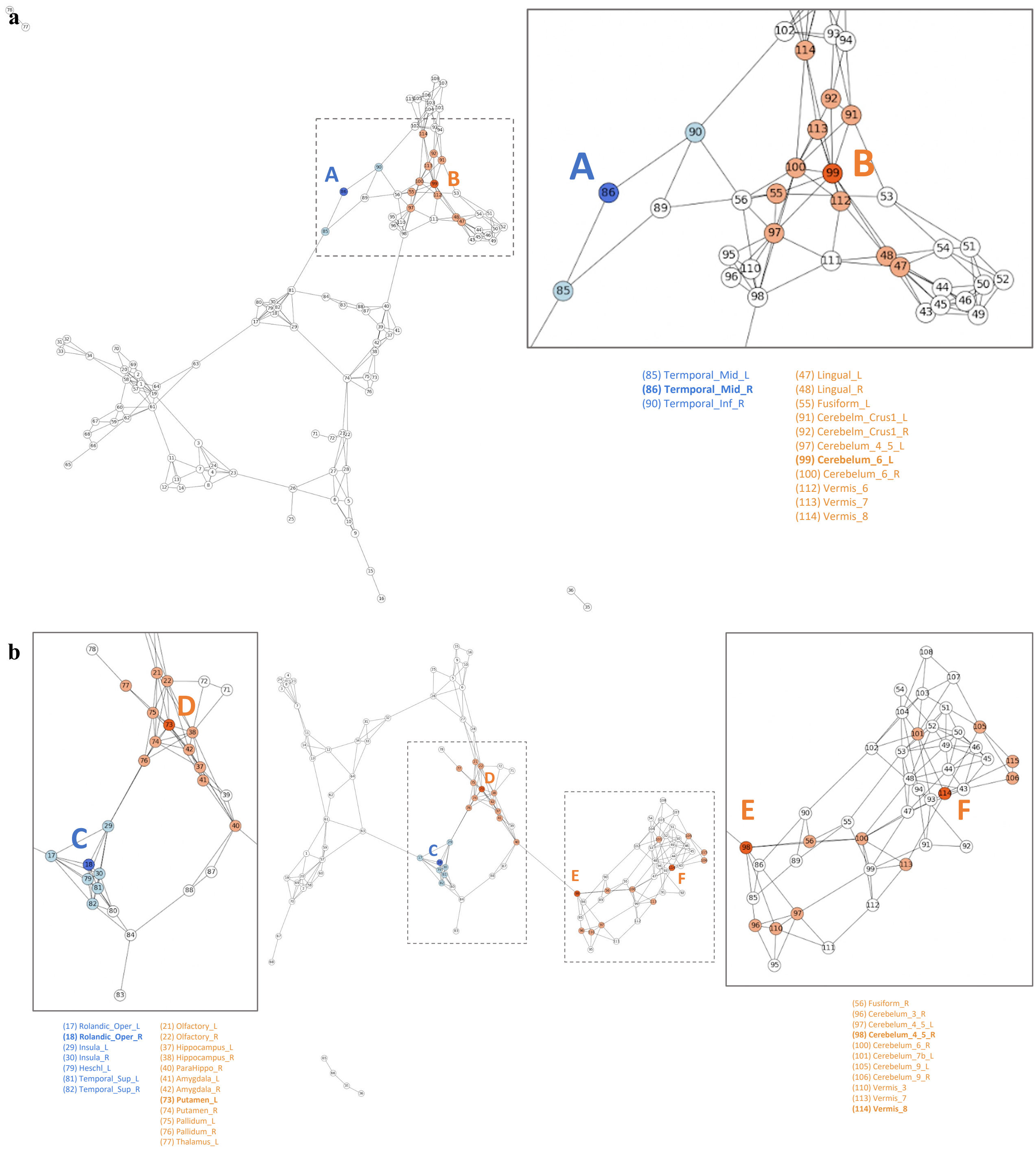

Figure S12. **(a)** AD group summary FCN and **(b)** LMCI group summary FCN.

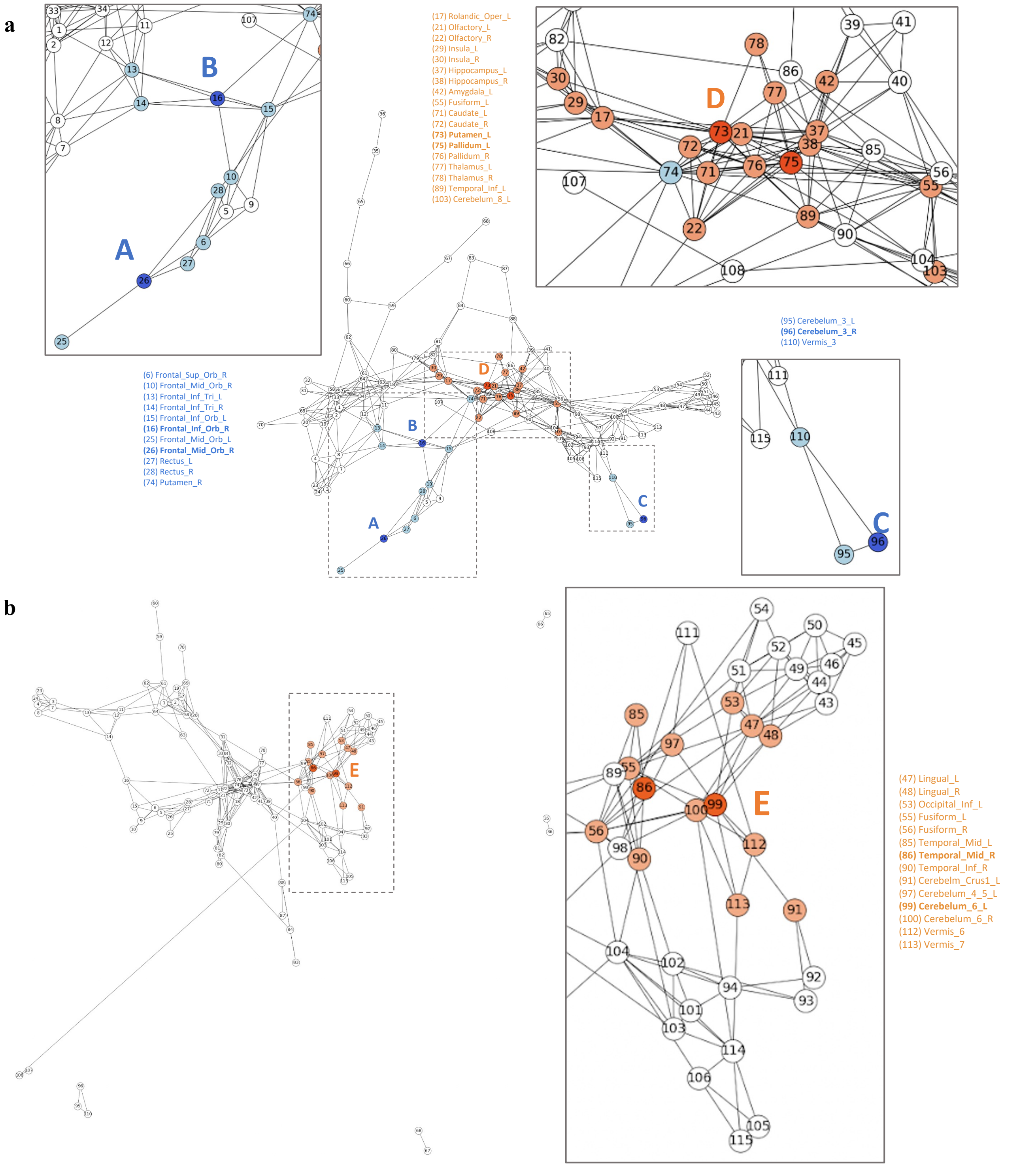

Figure S13. (**a**) EMCI group summary FCN and (**b**) LMCI group summary FCN.
